# Study of the Synergetic Effect of Co-Pyrolysis of Lignite and High-Density Polyethylene Aiming to Improve Utilization of Low-Rank Coal

**DOI:** 10.3390/polym13050759

**Published:** 2021-02-28

**Authors:** Ivan Kojić, Achim Bechtel, Nikoleta Aleksić, Dragana Životić, Snežana Trifunović, Gordana Gajica, Ksenija Stojanović

**Affiliations:** 1 Innovation Center of the Faculty of Chemistry, University of Belgrade, Studentski Trg 12-16, 11000 Belgrade, Serbia; ivankojic@chem.bg.ac.rs; 2 Department of Applied Geosciences and Geophysics, Montanuniversität Leoben, Peter-Tunner-Str. 5, A-8700 Leoben, Austria; achim.bechtel@outlook.de; 3 Faculty of Mining and Geology, University of Belgrade, Đušina 7, 11000 Belgrade, Serbia; nikoleta.aleksic@rgf.bg.ac.rs (N.A.); dragana.zivotic@rgf.bg.ac.rs (D.Ž.); 4 Faculty of Chemistry, University of Belgrade, Studentski Trg 12-16, 11000 Belgrade, Serbia; snezanat@chem.bg.ac.rs; 5 Institute of Chemistry, Technology and Metallurgy, Center of Chemistry, University of Belgrade, Studentski Trg 12-16, 11000 Belgrade, Serbia; ggajica@chem.bg.ac.rs

**Keywords:** lignite, HDPE, co-pyrolysis, synergetic effect

## Abstract

The mutual impact of low-quality lignite and high-density polyethylene (HDPE) during open system pyrolysis was investigated, aiming to improve utilization of lignite with simultaneous treatment of HDPE waste. Pyrolysis of lignite, HDPE, and their mixture (mass ratio, 1:1) was performed at temperatures 400, 450, 500, 550, and 600 °C. Initial substrates and pyrolysis products were characterized by thermogravimetric analysis (TGA), gas chromatography–mass spectrometry (GC–MS), specific carbon isotope analysis of individual hydrocarbons (δ^13^C), Rock-Eval pyrolysis, and elemental analysis. The positive synergetic effect during co-pyrolysis of lignite/HDPE mixture was observed at temperatures ≥450 °C, with the greatest being at 500 °C. The highest yield of liquid co-pyrolysis products with a similar composition to that of crude oils is also noticed at 500 °C. The yields of liquid and gaseous products and quality of pyrolytic products obtained by co-pyrolysis of lignite/HDPE mixture are notably improved compared with pyrolysis of lignite alone. On the other hand, data obtained from pyrolysis of HDPE alone indicate that it cannot be concurrent to well-developed catalytic thermal processes for polymer recycling. However, concerning the huge amount of produced HDPE, at least part of this plastic material can be reused for advanced thermal treatment of lignite, particularly in countries where this low-rank coal represents the main source of energy.

## 1. Introduction

Production of energy in Serbia is based on coal (50%), particularly lignite (92% of total coal resources) [1]. The main lignite deposits in Serbia are of Upper Miocene age. They are located in Kolubara and Kostolac basins, and in the Kovin deposit [2,3,4]. Although being a small country (about 7 million people in 2018), Serbia is placed among the 15 greatest producers of lignite [5]. Annually, Serbia produces about 38 Mt of lignite [6,7]. Therefore, it is of great importance to improve the rational utilization of lignite.

Coking coals are limited in rank to the interval of vitrinite reflectance from 1.00 to 1.40% [8]. Lignite and low-rank bituminous coal have huminite/vitrinite reflectance (Rr) in the range from 0.30 to 0.50% and cannot be used for coking. Our previous investigations showed that Serbian lignite is not suitable for coal briquetting, whereas a very limited number of samples possessed certain potential for fluidized bed gasification [2,9]. Most lignite is used in thermal power plants. In contrast to lignite consumption in thermal power plants, where lignite burns completely at high temperatures and where air pollution is controlled using electrostatic and bag-filtering dust precipitators, as well as flue gas desulphurization systems, the previous studies have shown that, owing to the low combustion temperature and low burning efficiency of the volatile matter in household stoves, pollutant emission factors from household coal combustion can be ~100 times higher than those from power plant coal boilers [10,11]. The recommendation from the World Health Organization (WHO) is the following: “*Unprocessed coal should not be used as a household fuel*” [12]. However, a relatively high amount of low-rank coal in addition to thermal power plants is used in households (for cooking and heating), particularly in Serbia, India, and China [13,14].

Numerous studies were performed on lignite pyrolysis as a method for its improved utilization [15,16,17]. A recent strategy in bioplastics development concerns the extensive use of natural products. Polysaccharides, pectin, and alginate represent the basic components in healable and safely dissolvable biocomposite that has potential applications in food packaging [18], whereas Solanyl^®^ type biopolymer was reinforced with lignocellulosic wood flour particles of small (0.07–0.15 mm) and medium size (0.30–0.50 mm) [19].

Recycling of plastics and conversion of polymeric materials into useful products (via pyrolysis, hydrolysis, hydrogenation, and gasification) on the large scale has occurred for more than 25 years. Regarding the recycling of hydrocarbon polymers, such as high-density polyethylene (HDPE), low-density polyethylene (LDPE), and polypropylene (PP), pyrolysis, particularly the catalytic cracking, gained notable attention [20,21,22,23]. Recently, pyrolysis was also used for recovery of caprolactam from teabag waste with simultaneous production of valuable solid and gas products [24].

The hydrocarbon generation potential of sedimentary organic matter (OM) depends on its quantity, quality/type, and maturity. The most important factor controlling the potential of OM of sedimentary rocks to produce liquid hydrocarbons is the amount of hydrogen in kerogen [25]. At the beginning of catagenesis, kerogen type I, and kerogen type II, which are characterized by a generally high hydrogen/carbon (H/C) ratio (i.e., Hydrogen Index), have greater liquid hydrocarbons generation potential than kerogen type III (depleted in hydrogen), representing the main kerogen type in humic coal [26]. In organic geochemical investigations, different pyrolytic methods are also widely used for estimation of the origin, type/quality, depositional environment, maturity, and hydrocarbon generation potential of sedimentary organic matter (OM) in the oil shale, coal, and source rocks [27,28,29,30,31].

In the last 15 years, much attention has been given to thermal treatment of coal/plastic blends. Namely, hydrocarbon plastic materials, PE, and PP can be the source of hydrogen during the pyrolysis of hydrogen depleted natural products such as coal and biomass. In this way, pyrolysis of mixtures of coal and plastic hydrocarbon materials (co-pyrolysis) results in a balance of carbon and hydrogen contents, giving the opportunity to certain advantages of the co-pyrolysis process [32,33]. Furthermore, the thermal decomposition of coal OM and most hydrocarbon-based polymers (HDPE, LDPE, and PP) occur in similar temperature ranges by free radical mechanism [34,35,36,37]. Therefore, chemical interactions between plastics- and coal thermal decomposition intermediates, during pyrolysis, may have a positive impact on the yield and quality of the obtained products. In previous studies, the synergetic effect (increase of experimental yield in relation to theoretical yield) during coal and plastics co-pyrolysis was mainly monitored by the thermogravimetric analysis [37,38] or, on the other hand, investigations were focused on a certain target product (e.g., production of hydrogen [39], or semi-coke [36]). As for coal and hydrocarbon fuels, formulas for the calculation of the calorific value of plastic waste material and biomass plastic fuel were proposed [40,41]. Less attention was given to detailed characterization of all three (solid, gas, and liquid) products obtained by co-pyrolysis of coal/plastic blends. This is particularly related to investigation of the molecular composition of liquid products, whereas, to the best of our knowledge, stable carbon isotopic measurements of individual compounds have never been performed. Furthermore, previous investigations were carried out on lignite or bituminous coal having a low ash (4–12%, dry basis) and relatively high carbon content (62–84%, dry, ash-free bases) [33,35,36], not on low-quality lignite with a high ash content (>40%) and, consequently, a low calorific value (net calorific value <15 MJ/kg). Our preliminary test on lignite having more than 30% of ash indicated the possible positive aspects of its co-pyrolysis with HDPE [42].

Therefore, the paper investigates lignite/HDPE co-pyrolysis as a possible method for utilization of lignite of extremely low quality (ash content > 40%; organic carbon content <30%). The objective was to examine and confirm the existence of a synergetic effect during low-quality lignite and HDPE co-pyrolysis, using a wide range of methods, and to establish the minimal co-pyrolysis temperature, which is required for a considerable synergetic effect. The manuscript also considers determination of the temperature corresponding to the highest yield of liquid co-pyrolysis products, having a composition comparable to that of crude oils. Although main attention is given to liquid co-pyrolysis products, characteristics of solid residues and gases obtained by lignite/HDPE co-pyrolysis were also studied and compared to those from pyrolysis of lignite and HDPE alone. Pyrolysis of lignite, HDPE, and their mixture (mass ratio, 1:1) was performed at various temperatures (400, 450, 500, 550, and 600 °C). For the characterization of initial substrates and pyrolysis products, and estimation of the synergetic effect, the following methods were used: thermogravimetric analysis (TGA), gas chromatography–mass spectrometry (GC–MS), specific carbon isotope analysis of individual hydrocarbons (δ^13^C), Rock-Eval pyrolysis, and elemental analysis. Detailed analysis of pyrolysis products and understanding the interactions between lignite OM and HDPE may contribute to the development of cost-effective and environmentally friendly processing of low-rank coal with simultaneous utilization of HDPE waste.

## 2. Materials and Methods

### 2.1. Samples

The experiments were performed on lignite from the Smederevsko Pomoravlje field (Kostolac Basin, Serbia). A short outline of the geological settings of the Kostolac Basin is given in the Supplementary Material, whereas a detailed description is given in previous publications [2,43,44,45]. The chosen lignite sample is immature (huminite reflectance, 0.30 ± 0.03%Rr) and has a low organic carbon content (29.77% dry basis; 56.30 dry, ash-free basis), low net calorific value (12.1 MJ/kg), and high ash content (47.12%), allowing to study the influence of HDPE on the low-quality lignite. Lignite was crushed and pulverized to <63 μm. Prior to pyrolysis experiments, extractable organic matter (bitumen) was removed, as explained in Section 2.2.

The plastic bag for food packing was used as a source of HDPE. The plastic bag was rinsed with *n*-hexane to remove possible impurities (e.g., dust), dried on air, and cut using scissors into small pieces. These pieces of HDPE were mixed with NaCl (extra pure, Zorka Pharma-Hemija d.o.o., Šabac, Serbia), transferred into ceramic mortar, and comminuted. NaCl served as an abrasive that crushes HDPE and prevents the sticking of HDPE particles. Larger pieces were further cut with scissors, and occasionally a new portion of NaCl was added. Then, mixture of HDPE particles and NaCl was additionally finely grounded in an agate mortar. The prepared mixture was carefully sieved through a 150 µm sieve. The obtained fraction <150 µm was dissolved in distilled water and filtered through a Büchner funnel. Powdered HDPE was rinsed with distilled water until negative reaction to chlorides (using AgNO_3_, PA grade, Centrohem, Stara Pazova, Serbia). After complete removal of NaCl, the powdered HDPE sample was air-dried. The preparation of samples was performed at the University of Belgrade, Faculty of Chemistry.

### 2.2. Extraction of Lignite

Extractable organic matter (EOM, bitumen) was extracted from the pulverized lignite sample using Soxhlet extraction with dichloromethane/methanol (both of HPLC grade, Carlo Erba Reagents, Val de Reuil, France) azeotrope (88:12, v:v) over 48 h. Solvent was removed by rotary vacuum evaporator. Bitumen was quantitatively transferred from the flask to small glass bottle using a Pasteur pipette and gently dried to the constant mass at ambient conditions. The lignite EOM was further treated in the same way as liquid pyrolysates (Section 2.5). The extraction was performed at the University of Belgrade, Faculty of Chemistry.

### 2.3. Thermogravimetric Analysis (TGA)

Thermogravimetric analysis (TGA) was performed on initial pre-extracted lignite, HDPE, and their mixture in the mass ratio of 1:1 using a Netzsch STA 449C instrument (Netzsch-Gerätebau GmbH, Selb, Germany). Samples were heated from 27 °C to 900 °C at a heating rate of 10 °C/min. The heating rate of 10 °C/min was chosen because, at high heating rates, especially for endothermic reactions, the furnace temperature increases faster than the sample temperature, resulting in error of measurement, particularly when analysis is carried-out in non-isothermal mode [46]. Nitrogen was used as the purge gas with the flow rate of 60 cm^3^/min. The flow rate of nitrogen was chosen to optimize the analysis of gases. Fourier transform infrared spectroscopy (FTIR) was applied for qualitative identification of generated gases, using ATI MATSON Infinity Series FTIR instrument (Labx, Midland, ON, Canada). TGA-FTIR analysis was done at the Montanuniversität Leoben, Department of Product Engineering.

### 2.4. Pyrolysis of Lignite, HDPE, and Their Mixture

**Figure 1:**
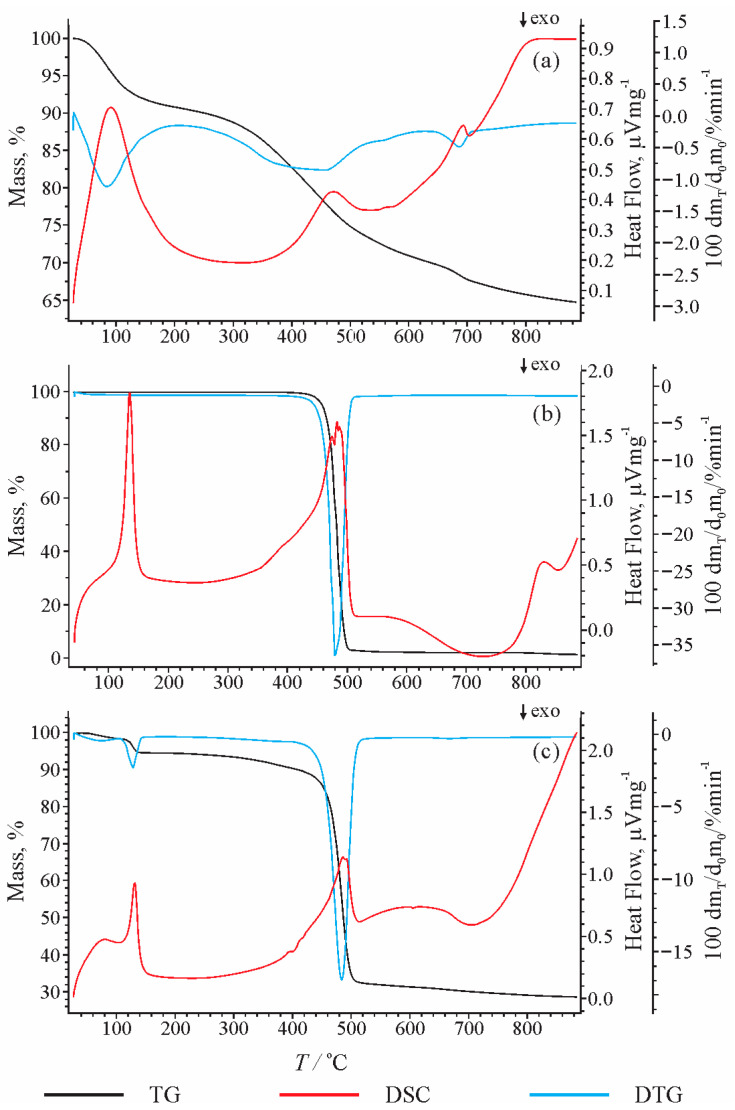
The characteristic weight loss (thermogravimetric analysis, TGA), differential scanning calorimetry (DSC), and derivative weight loss (DTG) curve of lignite (**a**), HDPE (**b**), and the lignite/HDPE mixture (**c**).

Pyrolysis of initial pre-extracted lignite, HDPE, and their mixture (mass ratio 1:1) was performed in an open pyrolysis system (Pyrolyser, Model MTF 10/15/130, Carbolite Limited, Parsons Lane, Hope, UK) at temperatures of 400, 450, 500, 550, and 600 °C under a nitrogen atmosphere. Particular attention was devoted to temperature range between 400 and 500 °C, concerning the thermogravimetric properties of initial lignite and HDPE, shown in Figure 1a,b, as well as the fact that coal exists in a plastic state under such conditions [34]. The lignite/HDPE mass ratio was chosen based on our preliminary studies and literature data, which indicated that the maximum synergetic effect is observed in mixtures containing between 20 and 50 wt.% of coal [35], as well as the report that a higher content of plastic can cause the formation of a thicker plastic film that avoids release of liquid pyrolysis products [38]. The samples were heated from 20 °C to the final pyrolysis temperature at a rate of 5 °C/min and the duration of all pyrolytic experiments at the final temperature was 4 h.

The furnace (15 cm long) was equipped with a quartz tube (inner diameter, 1 cm) that was 4.5 times longer than furnace. The quartz tube was situated within the furnace and equal parts of the quartz tube were outside of furnace. The tube was connected to the nitrogen supply at one side, while the other was connected to a trap (cooled to 0 °C), filled with 20 cm^3^ of chloroform, to which the pyrolysis products were transferred by the N_2_ flow. Liquid products were collected in cold trap, whereas released gases could not be collected. The sample (1 g) was situated in the quartz vessel and the vessel with the sample was transferred into the pyrolysis furnace in the middle of the quartz tube.

After cooling the pyrolytic system over night, the quartz tube was rinsed with chloroform (HPLC grade, Carlo Erba Reagents, Val de Reuil, France) and this extract was combined with the liquid product collected in the cold trap. Chloroform was removed by rotary vacuum evaporator. Liquid product was quantitatively transferred from the flask to small glass bottle using a Pasteur pipette and dried to the constant mass under ambient conditions. The solid residue was air-dried and weighed, whereas the yield of gas was calculated as follows: 100% − (yield of liquid pyrolysate + yield of solid residue). All experiments were repeated three times and average yields are reported here with standard deviations less than 0.5%. The experiments were done at the University of Belgrade, Faculty of Chemistry.

### 2.5. Analysis of Lignite EOM and Liquid Pyrolysis Products

Lignite EOM and liquid pyrolysis products were dissolved in chloroform, then quantitatively transferred to the flask by Pasteur pipette and left for solvent evaporation at room temperature. Samples were re-suspended in a small amount of dichloromethane (the mass ratio of sample and dichloromethane was 2:1.5) and then *n*-heptane (HPLC grade, Carlo Erba Reagents, Val de Reuil, France) was added (mass ratio *n*-heptane/lignite EOM or liquid pyrolysate = 40:1). The suspension was intensively shaken and left in a dark place for 16 h. The suspension was then refluxed for 20 min and filtered to separate asphaltenes. Asphaltene precipitate was rinsed several times with hot *n*-heptane for total removal of maltenes (*n*-heptane soluble fraction). Excess of *n*-heptane from filtrate (maltenes) was removed by rotary vacuum evaporator. Maltenes were quantitatively transferred from the flask to a small glass bottle using a Pasteur pipette and gently dried to the constant mass at ambient conditions. Maltenes from lignite EOM and the liquid pyrolysates were dissolved in the minimal volume of *n*-hexane (HPLC grade, Carlo Erba Reagents, Val de Reuil, France) (sonication in an ultrasonic bath) and separated into aliphatic, aromatic, and polar (NSO) fractions using micro-column chromatography. Silica gel 60 (Sigma Aldrich, St. Louis, MO, USA), previously activated at 180 °C for 8 h, was used as adsorbent (0.6 g SiO_2_ per 20 mg of sample). The aliphatic fraction was eluted with *n*-hexane, the aromatic fraction with mixture of *n*-hexane and dichloromethane (7:3, *v*:*v*), and the polar fraction with methanol/dichloromethane mixture (1:1, *v*:*v*). The fractions were collected in small glass bottles and gently dried to the constant mass at ambient conditions. Masses of fractions were measured and the percent of each fraction was determined. As GC–MS analysis showed that all three fractions (aliphatic, aromatic, and NSO) in liquid pyrolysates of HDPE comprise aliphatic hydrocarbons exclusively, they were combined and total liquid pyrolysate was injected into the GC–MS system.

The aliphatic and aromatic fractions from lignite and lignite–HDPE pyrolysates, as well as total liquid pyrolysates of HDPE, were analyzed by GC–MS technique, using an Agilent 7890A Gas Chromatograph (HP-5MS column, 30 m × 0.25 mm, 0.25 μm film thickness, carrier gas—helium with constant flow of 1.5 cm^3^/min), coupled to an Agilent 5975C Mass Selective Detector (Agilent Technologies, Santa Clara, CA, USA) (electron ionization energy of 70 eV). The following column temperature program was used: heating from 80 to 300 °C at a rate of 2 °C/min, isothermal at 300 °C for 20 min, then heating from 300 to 310 °C at a rate of 10 °C/min, and finally isothermal at 310 °C for 1 min. The injector temperature was 250 °C; the interface (transfer line) was kept at 280 °C; whereas temperatures of ion source and quadrupole were 230 °C and 150 °C, respectively. Samples were injected in split mode (1:5). The injection volume was 1 μL. GC–MS analysis was carried out in the scan mode. The mass spectrometer was operated in the electron ionization (EI) mode over a scan range from *m/z* 45 to *m/z* 550. The individual peaks were identified based on the mass spectra. The quantification of the compounds for calculating molecular parameters was performed by integrating peak areas using the ChemStation Enhanced Data Analysis Software (Agilent Technologies). The mentioned analyses were performed at the University of Belgrade, Faculty of Chemistry.

### 2.6. Specific Carbon Isotope Analysis of Individual Hydrocarbons in Lignite EOM and Liquid Pyrolysates

Specific carbon isotope analysis of individual hydrocarbons (δ^13^C) in the aliphatic fractions separated from lignite EOM and liquid pyrolysis products was performed using a Trace GC instrument attached to a ThermoFisher DELTA-V isotope ratio mass spectrometer via a combustion interface (GC Isolink, Thermo Fisher Scientific, Waltham, MA, USA). DB-5MS fused silica column (30 m length; i.d. 0.25 mm; 0.25 μm film thickness) was used. The oven temperature gradient was programmed from 70 °C to 300 °C at 4 °C/min, followed by an isothermal period of 15 min. Helium (flow 1.2 cm^3^/min) was used as carrier gas. For calibration, CO_2_ was injected at the beginning and end of each analysis. Stable isotope ratios are reported in delta notation (δ^13^C) [47] relative to the Vienna-Pee Dee Belemnite (V-PDB) standard (δ^13^C = [(^13^C/^12^C)sample/(^13^C/^12^C)standard − 1]). Delta notation is expressed in parts per thousand (‰). The analytical error was better than 0.2 ‰. The analysis was performed at the Montanuniversität Leoben, Department of Applied Geosciences and Geophysics.

### 2.7. Characterization of Initial Substrates and Solid Pyrolysis Products

Initial pre-extracted lignite; HDPE; and solid residues obtained by pyrolysis of lignite, HDPE, and their mixtures (Section 2.4.) were characterized by Rock-Eval and elemental analysis.

Rock-Eval pyrolysis [48] was performed using TOC/Rock-Eval-6 apparatus (Vinci Technologies, Nanterre, France). The sample aliquot was 7.3–12.8 mg. The IFP 160,000 standard was used for calibration. The programmed heating rate of both the pyrolysis and the oxidation ovens was 25 °C/min. Pyrolysis was done in the temperature range from 300 to 650 °C, while oxidation was performed up to 850 °C. The total time for sample analysis was 90 min. The entire process was managed using the software “RockSix” (Vinci Technologies, Nanterre, France). The analysis was carried out at the University of Belgrade, Faculty of Mining and Geology.

Elemental analysis was performed in order to determine the contents of total carbon, sulphur, nitrogen, hydrogen, and organic carbon (C_org_). Prior to determination of C_org_, carbonates were removed with diluted hydrochloric acid (1:3, *v*:*v*) from pre-extracted lignite and solid pyrolysis products of lignite and lignite/HDPE mixtures. The samples were analyzed using an Elementar Analysensysteme GmbH—Vario EL III CHNS/O Element Analyzer (Elementar Analysensysteme GmbH, Langenselbold, Germany). Ash yield measurements followed ISO 1171 (1997) [49]. Both analyses were performed at University of Belgrade, Faculty of Chemistry.

## 3. Results and Discussion

### 3.1. TGA Analysis

Figure 1a shows the TGA curve and derivative weight loss (DTG) curve of pre-extracted lignite. The first peak at 90.8 °C corresponds to loss of moisture. Degradation of OM starts at about 300 °C and the greatest weight loss is observed in the temperature interval between 400 and 500 °C, with the maximum at 471.2 °C. With further heating from 500 to 800 °C, the weight loss is less intense (<5% with temperature rise of 100 °C). The peak recorded at a temperature of 693 °C represents mostly mass loss caused by thermal alteration of minerals (clay and carbonate) [50]. The TGA curve shows that only about 25% of the lignite mass decomposes at 500 °C, while at 900 °C, it reaches 35%. The low degree of lignite degradation is in accordance with the low content of OM (total organic carbon of 29.77% and hydrogen of 2.79%; Section 3.3), as well as a high content of mineral matter (ash content of 47.12%), which is relatively stable and not subjected to significant decomposition even at a temperature >800 °C.

The TGA and DTG results of the plastic bag (Figure 1b) are consistent with the results of previous studies using other commercial HDPE sources [20,51]. The thermal decomposition of HDPE starts at about 430 °C, and almost complete degradation occurs at a temperature of about 500 °C. Differential scanning calorimetry (DSC) indicates the presence of two main peaks. The first peak at about 135 °C corresponds to the melting of HDPE, without loss of mass. The second peak, followed by weight loss, starts at about 390 °C and ends at about 505 °C. The presence of one sharp peak in DTG indicates only one degradation step of HDPE conversion. The temperature corresponding to the maximum release of volatile substances is 483.2 °C, while the largest weight loss, 35.28%, corresponds to a temperature of 479.5 °C. Comparison of the results from Figure 1a,b indicates that the intense decomposition of selected lignite and HDPE takes place in the same temperature range, from 400 to 500 °C, which was thus chosen for the study of the synergetic effect.

In the DSC curve of the lignite/HDPE mixture (Figure 1c), a wide peak at about 90 °C corresponds to the loss of moisture from lignite. In the presence of lignite, HDPE begins to melt at about a 5 °C lower temperature than during thermal decomposition of HDPE alone (Figure 1b,c). The result implies that release of moisture from the lignite that occurs before the melting of HDPE can accelerate the latter process. The temperature corresponding to onset of the most intense mass loss decreases from 474.7 °C (pure HDPE) to 464.9 °C in the presence of lignite, which is associated with slight widening of the peak. The greatest mass loss per minute is observed at 479.5 °C and 484.0 °C for the HDPE and lignite/HDPE mixture, respectively. The residual mass at 500 °C for lignite alone was 75%, and for HDPE alone was 4.79%, based on which the expected percentage of undecomposed residue for a mixture of lignite and HDPE (1:1) according to the theoretical calculation is (75% + 4.79%)/2 = 39.985%.

However, the experimental data showed that the percentage of undecomposed residue of a mixture of lignite/HDPE at 500 °C amounts to 33.42%, which is ~6% lower than the theoretical calculation (Figure 1). Previous studies reported a decrease of the experimental percentage of undecomposed residue in comparison with the theoretical value in the range of 2–4% [37,38]. The slightly greater reduction of undecomposed residue content observed here can be attributed to characteristics of initial immature lignite enriched in reactive –COOH and –OH groups. At a final temperature of 886 °C, the percent of undecomposed lignite and HDPE is 64.69% and 3.46%, respectively. The theoretically calculated percent of residual mass for the lignite/HDPE mixture at 886 °C is (64.69% + 3.46%)/2 = 34.075%, while the experimental one is 29.15% (Figure 1). Therefore, the difference between the theoretically calculated and experimental percent of residual mass at 886 °C and 500 °C is almost identical. This implies that, in the temperature range from 500 °C to 900 °C, there is almost no impact of HDPE on the lignite decomposition, as the experimental percentage of undecomposed residue remains about 5–6% lower than theoretical, as at 500 °C. This implies that the volatilisation processes of the lignite/HDPE mixture have been substantially finished at temperatures up to 500 °C.

### 3.2. The Yields of Pyrolysis Products Obtained by Open System Pyrolysis

The yields of pyrolysis products obtained by open system pyrolysis are given in Table 1.

As expected, increasing the temperature resulted in an increase of the conversion of all three substrates (lignite, HDPE, and their mixture) into liquid and gaseous products. However, the increase is much more prominent for HDPE and the lignite/HDPE mixture than for lignite alone. The greatest yield of liquid products for all three substrates is recorded at 500 °C, suggesting that, at higher temperatures, their further degradation into gas is favoured. The yield of gas obtained by co-pyrolysis of lignite/HDPE is obviously higher than the yield of gas obtained by pyrolysis of HDPE at 400, 450, and 500 °C, whereas it is opposite at 550 and 600 °C. The yield of gas is lower in the co-pyrolysis experiment lignite/HDPE in comparison with pyrolysis of lignite at 400 °C; at 450 °C, yields are comparable; whereas at temperatures of 500–600 °C, the yield of gas obtained from lignite/HDPE mixture is 1.7–1.9 higher than those from lignite. The yields of liquid products obtained by co-pyrolysis of lignite/HDPE are notably higher than by pyrolysis of lignite (12–15 times at temperatures ≥450 °C), and uniformly lower (for 2.4–8.5%) than by pyrolysis of HDPE. It is noteworthy that the yields of liquid and gaseous products obtained by co-pyrolysis of lignite/HDPE at 450 °C are two times higher than and similar to, respectively, those reported in literature for pyrolysis of lignite of better quality (74.05% of carbon dry, ash-free basis) at 550 °C [52], whereas our experiment at 500 °C provided two times greater yields of both liquid and gaseous products than in the cited reference. Co-pyrolysis of lignite/HDPE mixture at 450 and 500 °C gave higher yields of liquids and gases than pyrolysis of lignite of better quality (64.35% of carbon, dry basis, and net calorific value of 25.78 MJ/kg) at 600 °C [16]. The yields of liquid and gaseous products of lignite/HDPE co-pyrolysis at 500 °C are also higher than the yields of these products obtained by mild liquefaction of lignite (temperature of 450 °C and pressure of 11.5 MPa in the presence of solvent, catalyst, and hydrogen) [17], which contained nearly two times higher amount of carbon than lignite used in the current study. On the other hand, yields of liquid products obtained from pyrolysis of HDPE alone indicate that it cannot be concurrent to well-developed catalytic pyrolytic processes ([22] and references therein). However, concerning the huge amount of produced HDPE (the third largest produced plastic material in the world, after polyvinylchloride and polypropylene in terms of volume), at least part of it can be used for improved utilization of lignite, particularly in countries where this low-rank coal represents the main energy source.

The evaluation of the interactions between lignite and HDPE wascarried out by comparing the experimental yields of the pyrolysis of the mixture lignite/HDPE and theoretical (calculated) yields = (yield of lignite pyrolysis + yield HDPE pyrolysis)/2 (Table 1). At a temperature of 400 °C, the experimental and theoretical yields are very similar, indicating almost no synergetic effect between lignite and HDPE. This result can be attributed to the relatively high thermal stability of HDPE up to 400 °C, which is then followed by an abrupt single mass loss step between 400 and 500 °C (Figure 1b). At temperatures >450 °C, experimental yields of liquid and gas products are obviously higher than theoretical yields (Table 1), indicating a synergetic effect between lignite and HDPE. At a temperature of 450 °C, the difference between the experimental yields and theoretical yields is greater for liquid products than for gases, whereas at ≥500 °C, the result is inverse, which is consistent with prevalent kerogen type III in lignite, and generally more intense degradation at higher temperatures. The most intense synergetic effect between lignite and HDPE is detected at 500 °C, and reduces with further heating. The elemental analysis and calculated calorific values of solid residues of lignite/HDPE mixtures (Section 3.3.) also indicate no influence with heating above 500 °C. The mentioned results are consistent with TGA data, which showed that, at temperatures >500 °C, there is almost no impact of HDPE on the lignite decomposition. Because the manuscript is focused on the lignite/HDPE synergetic effect with particular attention devoted to liquid and solid products obtained by co-pyrolysis, specific analyses (GC–MS, Rock-Eval, carbon isotope measurements) were performed on products obtained at 400, 450, and 500 °C.

### 3.3. Rock Eval Data and Elemental Analysis

**Figure 2:**
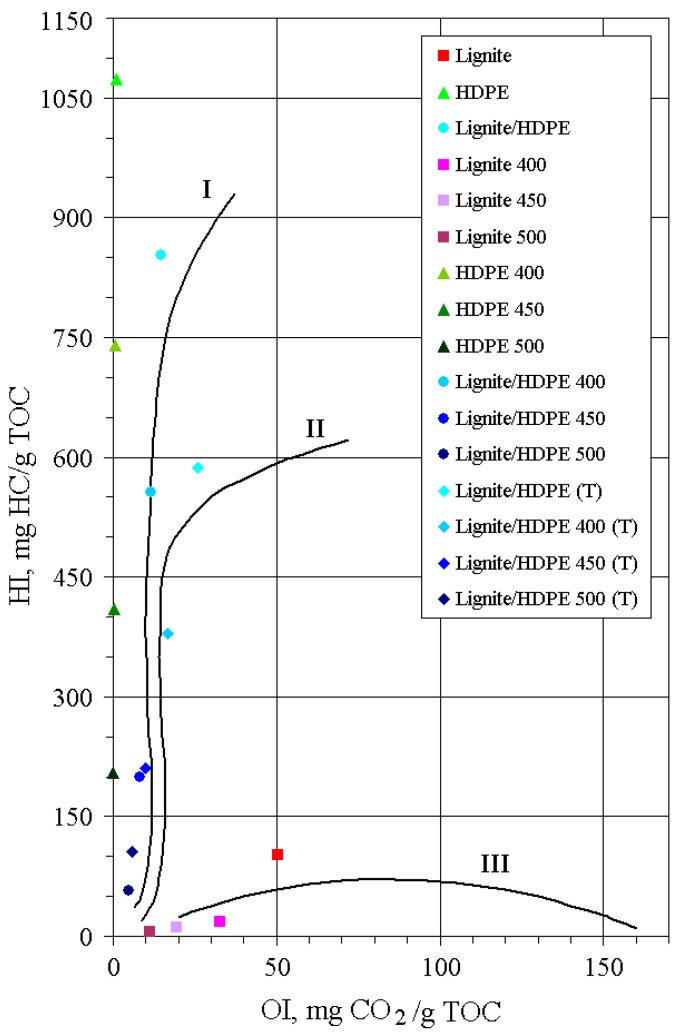
The hydrogen index (HI) vs. oxygen index (OI) plot. **Legend:** TOC—total organic carbon.

The results of Rock-Eval pyrolysis comprising S1, S2, S3, T_max_, total organic carbon (TOC), hydrogen index (HI), oxygen index (OI), and production index (PI) are listed in Table 2. As expected, the HI versus OI diagram (Figure 2) clearly indicates kerogen type III for lignite, and very high liquid generation potential for HDPE exceeding those of immature kerogen type I [26]. The lignite/HDPE mixture also demonstrated a high liquid generation potential corresponding to kerogen type I. The solid residues of all substrates obtained by the open system pyrolysis show a decreasing trend for HI and OI with the increase of pyrolysis temperature as a result of generation of liquid and gaseous products. An abrupt decrease of HI for HDPE and lignite/HDPE residues between 400 and 500 °C (Table 2) is consistent with the greatest mass loss in the same temperature range, detected by TGA (Figure 1).

**Figure 3:**
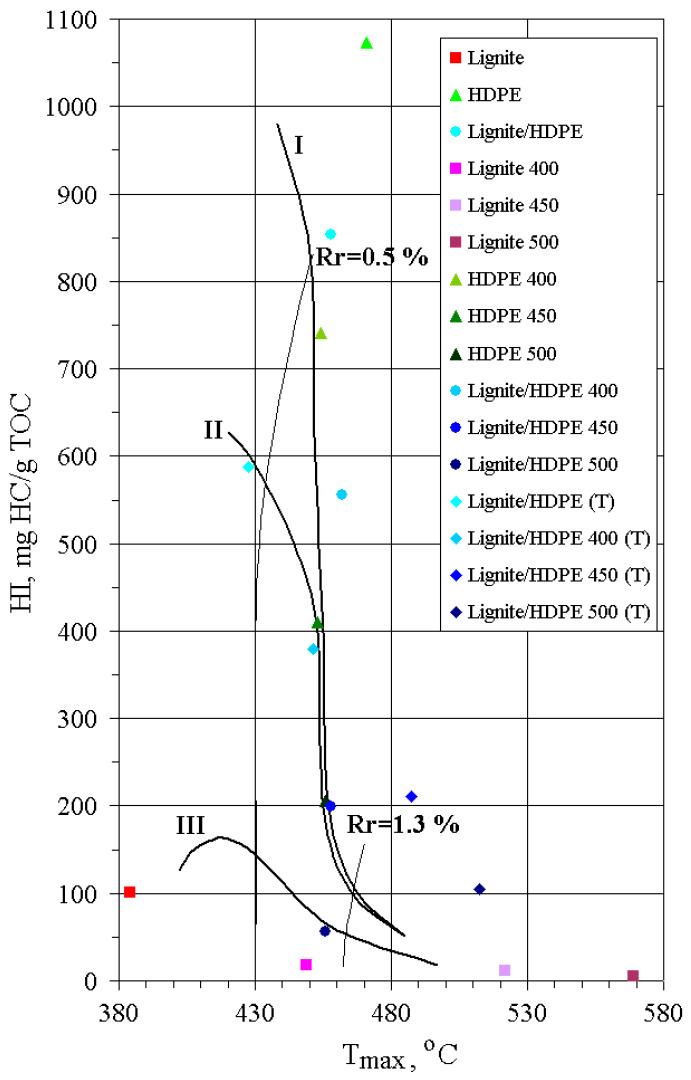
The HI vs. T_max_ plot.

The content of TOC increases in all substrates with the increase of pyrolysis temperature, implying that, during thermal decomposition, aliphatic chains enriched in hydrogen are preferably released, whereas solid residues become enriched in carbon. T_max_ and PI of solid residues increase with temperature, indicating an increase of OM maturity (Table 2; Figure 3). However, this rise is much more pronounced for lignite than for HDPE and lignite/HDPE mixtures owing to the abrupt aromatization of lignite OM. On the other hand, similar T_max_ temperatures for solid residues of HDPE and lignite/HDPE mixtures indicate that decomposition of HDPE is not accompanied with aromatization; rather, a waxy paraffinic residue is formed.

The experimental values of Rock-Eval parameters (S1, S2, and HI, which indicate hydrocarbon generation potential) of the lignite/HDPE mixture are higher than the theoretical values, (lignite + HDPE)/2, for initial non-heated substrates. On the other hand, the experimental values of the mentioned Rock-Eval parameters of the lignite/HDPE mixture are lower than the theoretical ones for solid residues obtained by pyrolysis at 450 and 500 °C (Table 2). The results are in concordance with those from open system pyrolysis (Table 1), indicating the synergetic effect between lignite and HDPE at the aforementioned temperatures, associated with greater production of liquid and gas.

The content of organic carbon (C_org_) obtained by elemental analysis is almost identical to the TOC from Rock Eval pyrolysis (Table 2 and Table 3). The notable increase of TOC (C_org_) in solid residues of lignite/HDPE mixtures in comparison with solid residues of lignite and initial lignite has a positive impact on the calorific value (Table 4). In accordance with HI (Table 2), the content of hydrogen shows the following trend: HDPE > lignite/HDPE mixture > lignite, and decreases in all substrates with the rise in temperature (Table 3). The content of oxygen also shows a decreasing trend with the rise in temperature (Table 3), resulting from defunctionalization reactions of lignite type III kerogen, enriched in carboxylic, hydroxyl, and carbonyl groups.

Concerning an environmental impact, the substantial feature of solid residues obtained by co-pyrolysis of lignite/HDPE mixture is a lower amount of sulphur than in initial lignite and its solid pyrolysis residues. The content of sulphur continuously decreased with the increase of pyrolysis temperature (Table 3), most probably due to the higher reactivity of C–S in comparison with C–C bonds. The theoretical (calculated) values and experimental results of elemental analysis are almost identical for untreated mixture lignite/HDPE and very similar for residues obtained at 400 °C. On the other hand, the increase of carbon content associated with a double decrease of hydrogen content is evident for experimental data of lignite/HDPE mixture residues obtained at temperatures ≥450 °C in comparison with theoretical ones, confirming the impact of the synergetic effect.

Net calorific values of lignite and lignite/HDPE solid residues calculated based on 10 formulas [40,41,53,54,55,56,57,58,59] proposed in the literature are listed in Table 4. Net calorific value of lignite residues continuously increases with the rise in temperature, whereas for lignite/HDPE mixture residues, the influence of pyrolysis temperature was less pronounced, and the greatest values are detected at 500 °C. Net calorific values of lignite/HDPE mixture residues, ranging from 22.87 to 27.48 MJ/kg, are notably higher than those of initial lignite and lignite residues (9.73–15.62 MJ/kg), as well as than the calorific value of char obtained by fast pyrolysis of HDPE waste at 450 °C, reported in the literature (18.83 MJ/kg, [60]). Furthermore, net calorific values of lignite/HDPE solid residues are greater compared with lignite (7–20 MJ/kg), and similar to sub-bituminous coal (19–26 MJ/kg) and high-volatile bituminous coal (24–35 MJ/kg) [61,62]. In comparison with solid residues obtained by HDPE pyrolysis, co-pyrolysis solid products have better combustion properties, as lignite has a stabilizing effect on the plastic residues morphology and prevents its melting [63]. Containing a relatively high amount of carbon (Table 3) and clay (from the lignite mineral matter [2]), the solid residues obtained by lignite/HDPE pyrolysis, in addition to direct combustion, may serve for adsorption of different pollutants from waste water; this would be the objective of our further investigations.

### 3.4. Characteristics of Liquid Pyrolysates

The bulk composition of EOM of initial lignite and liquid pyrolysis products is shown in Table 5. The EOM of lignite is notably dominated by polar, NSO compounds, and asphaltenes, typical for immature terrestrial OM. The liquid products obtained by pyrolysis of lignite also have a relatively low percent of hydrocarbons that does not exceed 31%. The liquid pyrolysates of HDPE at all temperatures consist of aliphatic hydrocarbons only. The liquid products obtained by lignite/HDPE co-pyrolysis have ~2.3 times higher content of hydrocarbons than corresponding liquid pyrolysates of lignite, indicating an improvement in composition. The contents of hydrocarbons in lignite/HDPE pyrolysates are higher than the theoretical ones at temperatures ≥450 °C, where the synergetic effect was observed (Table 5).

Furthermore, the content of hydrocarbons in lignite/HDPE pyrolysates obtained at 450 and 500 °C is similar or even higher than in crude oils. HDPE serves as a hydrogen donor to lignite OM, which is a clue for the generation of liquid hydrocarbons. This is also evident from eight times higher liquid hydrocarbon generation potential, expressed by HI from Rock-Eval, of the lignite/HDPE mixture in comparison with lignite (Table 2). HDPE also serves as a solvent, because it melts at about 135 °C and, in the temperature range between 450 and 500 °C, it is in a liquid state. Decomposition of both lignite and HDPE under applied pyrolysis conditions was carried out via free radical mechanism. Radicals formed from lignite kerogen can be easily stabilized in the presence of HDPE, capturing the H radicals from HDPE. This prevents the secondary processes (further cracking, coking on carbon residue) and contributes to increasing the content of liquid hydrocarbons. Simultaneously, capture of hydrogen radicals by lignite OM results in the formation of new reactive radical places in HDPE that promotes further cracking of polymer. Because different zeolite catalysts (e.g., clinoptilolite, HZSM-5) showed a positive impact on HDPE pyrolysis [64], the certain influence of clays that prevail in lignite mineral matter [2] on thermal decomposition of HDPE cannot be excluded. Termination reactions between radicals formed from kerogen and HDPE also occur. Furthermore, longer radicals can be disproportionated into alkene (Table 6 and Table 7) and new reactive radical.

Diterpenoids prevail in the aliphatic and aromatic fraction of lignite EOM. The main constituents of aliphatic fraction of all liquid pyrolysates at each temperature are *n*-alkanes and terminal *n*-alkenes, having similar distributions (Figures S1–S3; Table 6). Aliphatic fractions of lignite pyrolysates contain diterpenoids (pimarane, 16α(H)-phyllocladane), isoprenoids (pristene, norpristane, pristane, and phytane), and hopanes, whereas in liquid pyrolysates of lignite/HDPE, these biomarkers cannot be identified owing to the extremely low concentrations, even using their typical fragmentation ions *m/z* 123, 183, and 191, respectively. In addition to *n*-alkanes and terminal *n*-alkenes, liquid pyrolysates of HDPE and lignite/HDPE mixtures contain terminal *n*-dienes.

In the liquid pyrolysates of lignite, *n*-alkanes prevail over terminal *n*-alkenes; in pyrolysates of HDPE, the opposite trend is observed; whereas in pyrolysates of lignite/HDPE, they are present in very similar amounts. With the increase of temperature pyrolysis, the ratio of *n*-alkenes to *n*-alkanes slightly increases, consistent with data from the literature [65], implying more intense cracking and further disproportionation of the formed radicals (Table 7; Figures S1–S3).

In contrast to initial lignite EOM, which is characterized by notable predominance of long-chain odd *n*-alkane homologues (carbon preference index [66], CPI > 4), aliphatic fractions of all liquid pyrolysates display uniform distributions of odd and even *n*-alkane and *n*-alkene homologues (CPI ~1; Table 7; Figures S1–S3). Pyrolysates of lignite obtained at all temperatures are characterized by dominance of mid-chain C_21_-C_25_ normal hydrocarbon homologues, indicating almost no impact of temperature on *n*-alkane and *n*-alkene distributions (Figure S1; Table 6). Pyrolysates of HDPE and lignite/HDPE mixtures obtained at 400 °C have higher abundance of long chain-homologues with a broad maximum in the C_24_ to C_32_ range. This prevalence became slightly less prominent in pyrolysates of HDPE and lignite/HDPE mixtures at 450 °C, whereas in pyrolysate of HDPE and co-pyrolysate of lignite/HDPE at 500 °C, the increase of short-chain homologues is evident (Figures S2 and S3; Table 6).

Among the present hydrocarbons with a normal skeleton (alkanes, alkenes, and dienes), *n*-alkane series can be the most accurately separated from alkenes and dienes, using the typical ion fragmentogram *m/z* 71 (Figure S4) that enabled precise peak integration up to C_33_. Therefore, for more detailed interpretation of lignite/HDPE interactions, typical geochemical parameters based on distributions of *n*-alkanes were used (Table 7). Liquid pyrolysates of lignite at all three temperatures have almost equal distributions of *n*-alkanes with prevalence of mid-chain homologues. In all three HDPE liquid pyrolysates, domination of long-chain homologues is observed; however, with the increase in temperature, the decrease in abundance of long-chain homologues associated with the increase of short-chain *n*-alkanes and without a change in the content of mid-chain homologues is noticed (Table 7).

**Table 7:** Values of Σ*n*-alk-1-enes/Σ*n*-alkanes ratio and values of common organic geochemical parameters based on distributions of *n*-alkanes in lignite EOM and the liquid products obtained in open system pyrolysis.

Sample	Σ*n*-alk-1-enes/Σ*n*-alkanes	CPI	*n*-C_15_ − *n*-C_20_ (%)	*n*-C_21_ − *n*-C_25_ (%)	*n*-C_26_ − *n*-C_33_ (%)	*n*-C_17_/*n*-C_27_
Lignite EOM	N.D.	4.61	1	17	82	0.003
Lignite 400	0.38	1.12	23	49	28	0.14
Lignite 450	0.41	1.08	24	45	31	0.26
Lignite 500	0.64	1.05	24	48	28	0.29
HDPE 400	1.25	1.03	27	24	49	0.93
HDPE 450	1.37	0.96	32	23	45	1.17
HDPE 500	1.47	0.95	37	24	39	1.53
Lignite/HDPE 400	0.93	1.00	24	27	49	0.83
Lignite/HDPE 450	0.96	1.03	25	26	49	0.82
Lignite/HDPE 500	0.97	1.02	39	23	38	1.39

**Legend**: N.D.—not determined owing to the absence of *n*-alk-1-enes in lignite EOM (Figure S1a); CPI—carbon preference index determined for distribution of *n*-alkanes C_23_-C_33_, CPI = 1/2 × [Σodd(*n*-C_23_ − *n*-C_33_)/Σeven(*n*-C_22_ − *n*-C_32_) + Σodd(*n*-C_23_ − *n*-C_33_)/Σeven(*n*-C_24_ − *n*-C_34_)] [66]; *n*-C_15_ − *n*-C_20_ (%) = (Σ *n*-C_15_ − *n*-C_20_) x 100/Σ of total (*n*-C_15_ − *n*-C_33_) *n*-alkanes; *n*-C_21_ − *n*-C_25_ (%) = (Σ *n*-C_21_ − *n*-C_25_) × 100/Σ of total (*n*-C_15_ − *n*-C_33_) *n*-alkanes; *n*-C_26_ − *n*-C_33_ (%) = (Σ *n*-C_26_ − *n*-C_33_) × 100/Σ of total (*n*-C_15_ − *n*-C_33_) *n*-alkanes; C_x_ designates *n*-alkane and x represents total number of carbon atoms; Σ*n*-alk-1-enes/Σ*n*-alkanes ratio is calculated from total ion chromatograms (TICs) of aliphatic fractions; *n*-alkane parameters are calculated from the typical *m/z* 71 mass fragmentogram of aliphatic fraction (Figure S4).

Liquid pyrolysate of lignite HDPE/mixture at 400 °C has an almost identical composition of *n*-alkanes to pyrolysate of HDPE at this temperature, consistent with a much higher impact of HDPE than lignite in liquid pyrolysate (Table 1), confirming no significant interaction between lignite OM and HDPE at 400 °C. In lignite/HDPE liquid pyrolysate at 450 °C, the distribution of *n*-alkanes is still similar to those in HDPE pyrolysate at the same temperature, although the former has a slightly elevated content of long-chain homologues and lower amount of short-chain homologues. In comparison with lignite pyrolysate at 450 °C, lignite/HDPE pyrolysate has a higher content of long-chain homologues and proportionally lower amount of mid *n*-alkanes. The mentioned data indicate that, at 450 °C, the interaction between lignite and HDPE resulted in more intense release of long-chain homologues. This is consistent with the observation that lower molecular weight units of the normal chain are more stable than the initial polymer [67] and immature kerogen [26]. At a temperature of 500 °C, the greater percent of short-chain *n*-alkanes in lignite/HDPE pyrolysate is detected than in both lignite and HDPE pyrolysate (Table 7; Figures S1d, S2c, and S3c).

**Figure 4:**
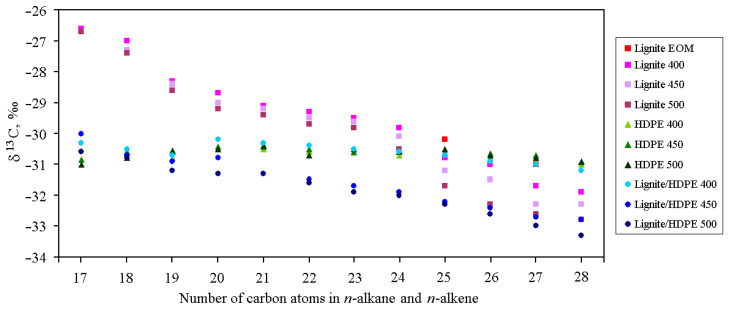
Carbon isotopic compositions (δ^13^C) of individual (*n*-alkane + *n*-alk-1-ene) doublets in aliphatic fractions of lignite EOM and liquid products obtained by pyrolysis of lignite, HDPE, and their mixtures (1:1) in the open system at 400 °C, 450 °C, and 500 °C. For homologues ≥C_26_, terminal dienes are also included, as they co-eluted with *n*-alk-1-enes.

The interactions between lignite OM and HDPE were further evaluated by measurement of the stable carbon isotope composition of individual *n*-alkane/*n*-alkene pairs (sufficient precise separation of their peaks was not possible in total ion chromatograms (TICs), even manually, for that analysis), which represented the most abundant compounds in all liquid pyrolysis products (Figures S1–S3). The results are presented in Figure 4.

Homologues up to C_28_ have been involved in interpretation, owing to the possible impact of hopanes on δ^13^C of higher homologues in lignite pyrolysates (Figure S1). δ^13^C values of normal hydrocarbons in lignite pyrolysates ranging from −26.6 to −32.8‰ are typical for precursor lipids of C3-terrestrial plants [68], showing z decreasing trend with chain length in all samples, as usual for coals [2,69,70,71]. Long-chain homologues exhibit slight enrichment in ^12^C with temperature increase, whereas this change was less pronounced for short-chain counterparts.

δ^13^C values of individual normal hydrocarbons in HDPE pyrolysates are quite uniform, ranging from −30.5‰ to −31.0‰, exhibiting almost no change with temperature (Figure 4). This is in accordance with their origin from polymer material, not from natural sedimentary OM, where short-, mid-, and long-chain homologues have different precursors [26]. Consistent with very similar distributions of normal hydrocarbons in liquid pyrolysates of HDPE and lignite/HDPE mixture at 400 °C (Figures S2a and S3a), their δ^13^C signatures also reveal notable similarity (Figure 4). In pyrolysates of lignite/HDPE mixture at 450 °C, δ^13^C values of short-chain normal hydrocarbons are still much closer to those of HDPE pyrolysate than lignite pyrolysate, signifying their prevalent origin from HDPE, whereas δ^13^C values of mid- and long-chain homologues are more negative than in both lignite pyrolysate and HDPE pyrolysate. In lignite/HDPE pyrolysate at 500 °C, this effect of enrichment of normal hydrocarbons in ^12^C isotope (in comparison with lignite and HDPE pyrolysate) is evident in the whole range C_17_–C_28_ (Figure 4). Data confirm the interaction between lignite and HDPE at 450 °C and particularly at 500 °C, which promotes degradation of both HDPE and kerogen, associated with preferred cleavage of more labile ^12^C–^12^C bonds.

Liquid pyrolysates of HDPE do not contain aromatic hydrocarbons; *n*-alkylbenzenes were identified in traces via characteristic *m/z* 91 mass fragmentogram, however, in an insufficient quantity for integration and interpretation. Solely compounds present in lignite extracts and lignite pyrolysis products are cadalene and retene (Figure S5, Table 8).

Aromatic fractions of lignite and lignite/HDPE pyrolysates consist of naphthalene, phenanthrene, fluorene, pyrene, chrysene, dibenzofuran, and their methylated derivatives (Figures S5 and S6, Table 8). As expected, an increase of pyrolysis temperature resulted in a rise of common geochemical maturity parameters calculated from distributions of naphthalene and phenanthrene derivatives in lignite pyrolysates (Table 9) [72,73,74,75,76]. The exception is MPI 1 (containing unsubstituted phenanthrene in the denominator), which showed an opposite trend. This can be attributed to preferable releasing of unsubstituted aromatics from lignite, rather than their methylated counterparts, with the increase of pyrolysis temperature, which is documented by an apparent decrease of the PAI 1 ratio from 400 °C to 500 °C (Table 9). Although HDPE pyrolysates do not contain aromatic compounds detected in lignite and its pyrolysates, differences in TICs of aromatic fractions of lignite and lignite/HDPE pyrolysates are evident (Figures S5 and S6; Table 8). This is reflected through the variations in contents of phenanthrene relative to its methylated derivatives, as well as the phenanthrene to cadalene ratio, assuring the influence of HDPE on cracking of lignite kerogen. The impact of HDPE on distributions of aromatic hydrocarbons derived from lignite kerogen cracking is also indicative based on the differences in values of geochemical parameters (Table 9; Figures S5–S7). In accordance with the weak impact of HDPE on lignite OM at 400 °C, differences in geochemical ratios are negligible, whereas they become more evident with the temperature increase, confirming the synergetic effect during lignite/HDPE co-pyrolysis at 450 °C and 500 °C.

The compositions of individual hydrocarbons in the aliphatic and the aromatic fractions of liquid lignite/HDPE co-pyrolysis product at 500 °C are similar to those of higher rank coal and crude oils of terrestrial origin. The main difference between the compositions of liquid lignite/HDPE pyrolysate and crude oil is reflected through the presence of *n*-alkenes and *n*-dienes in pyrolysate (Figure S3; Table 6), which are absent in crude oil. However, the presence of these unsaturated hydrocarbons in liquid co-pyrolysate does not represent a significant problem, as these hydrocarbons can be more easily converted into branched and cyclic hydrocarbons than *n*-alkanes, via reforming processes, giving an opportunity for producing high octane rating gasoline. On the other hand, terminal *n*-alkenes can be separated and used as chemical feedstock for plastic and detergent manufacture [77].

### 3.5. The Composition of Gaseous Products

**Figure 5:**
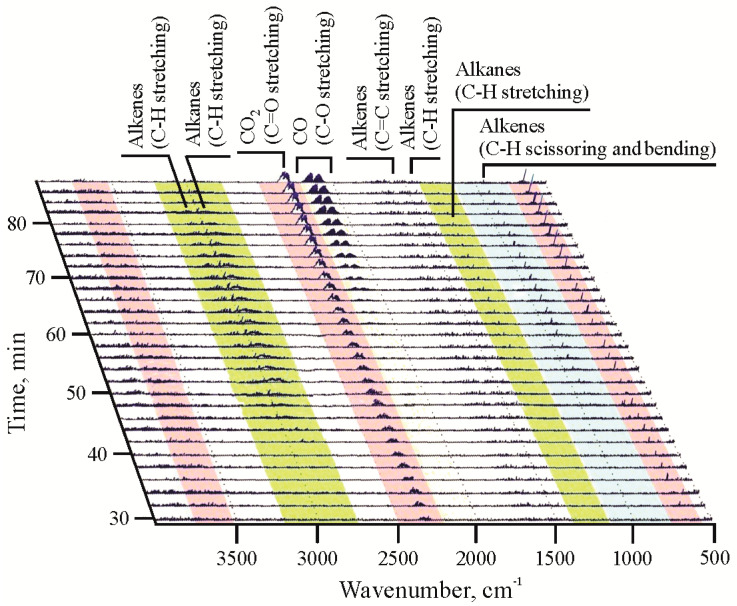
Enlarged image of thermogravimetric analysis (TGA)-Fourier transform infrared spectroscopy (FTIR) thermogram of lignite/HDPE mixture with indicated peaks. A time of 20 min corresponds to a temperature of 227 °C, 40 min to 427 °C, 60 min to 627 °C, and 80 min to 827 °C. The ramp rate was 10 °C/min (full TGA-FTIR thermograms of lignite, HDPE, and lignite/HDPE mixture are shown in Figure S8).

Gaseous products produced in the open system pyrolysis could not be collected and analyzed. The qualitative composition of gases was, therefore, estimated based on TGA coupled to Fourier transform infrared spectroscopy (FTIR) [78,79] (Figure 5 and Figure S8). However, it should be mentioned that certain differences in the composition of gases obtained by TGA and the open system pyrolysis are expected as it depends on pyrolysis conditions and the heating rate.

The TGA-FTIR thermogram of lignite (Figure S8a) indicates that the produced gas mainly consists of water vapour (O–H stretching peaks around 3700 cm^−1^ and O–H bending peaks from 1400 to 1700 cm^−1^), CO_2_ (peaks between 2300 cm^−1^ and 2400 cm^−1^ corresponding to C=O stretching), and CO (peaks between 2100 cm^−1^ and 2200 cm^−1^ representing C–O stretching). Peaks corresponding to water vapour are visible at 100 °C, indicating the removal of moisture from lignite, whereas peaks in the temperature range from 420 °C to 620 °C correspond to removal of H_2_O by dehydration of lignite OM (e.g., alcohol groups) and clay minerals, which represent the main inorganic constituents of lignite [2]. Release of CO_2_ starts above 220 °C and can be attributed to decarboxylation reactions of lignite OM, which is rich in carboxylic groups, whereas CO_2_ formed at temperatures >650 °C corresponds to degradation of carbonates. Release of CO starts at about 500 °C, being the most pronounced at around 700 °C. This reflects decarbonylation reactions, but CO also can be formed by reaction between CO_2_ and a small amount of generated hydrocarbons at high temperatures. Hydrocarbons in lignite gas are observed in traces only, and they are represented mainly by gaseous alkenes, as documented by very small peaks at around 3100 cm^−1^, corresponding to C–H stretching; around 1650 cm^−1^ corresponding to C=C stretching; and around 950 cm^−1^, corresponding to C–H bending (Figure S8a).

The main components in the gaseous product of HDPE decomposition are light hydrocarbons (alkanes and alkenes). Gaseous alkanes are characterized by peaks around 2950 cm^−1^, representing C–H stretching, and peaks around 1370 cm^−1^, representing C–H scissoring and bending. Gaseous alkenes are represented by peaks at around 3100 cm^−1^, corresponding to C–H stretching; peaks around 1450 cm^−1^, corresponding to C=C stretching; as well as by peaks observed around 950 cm^−1^, corresponding to C–H bending (Figure S8b). More pronounced peaks of C–H than of C–C or C=C vibrations indicate that main gaseous products are methane and ethene. The intense releasing of hydrocarbons begins at a temperature higher than 450 °C. A small amount of produced CO_2_ (peaks between 2300 cm^−1^ and 2400 cm^−1^ corresponding to C=O stretching) can be explained by the presence of minor amount of oxygen in initial HDPE [42], most probably originating from additives that are intercalated to improve the properties of HDPE [80]. Further reaction of released CO_2_ with hydrocarbons at high temperatures (above 850 °C) resulted in the formation of CO, whose presence is documented by C–O stretching peaks between 2100 cm^−1^ and 2200 cm^−1^ (Figure S8b). The obtained result suggests that pyrolysis of HDPE produces a valuable gaseous product rich in hydrocarbons.

TGA-FTIR thermogram of lignite/HDPE mixture reveals that gas contains all of the above-mentioned components (light hydrocarbons, CO_2_, CO, and water vapour) detected in TGA-FTIR thermograms of pure lignite and pure HDPE, with prevalence of CO_2_ and CO (Figure 5 and Figure S8c). The obtained results suggest that the presence of HDPE can significantly improve the quality of the gas composition in comparison with gas produced from lignite alone, which is primarily reflected through the elevated content of hydrocarbons. Although TGA-FTIR thermograms enable the qualitative gas composition assessment only, the obtained results are in concordance with more precise published results of GC analysis of gaseous products obtained by pyrolysis of coal, HDPE, and their mixture [35,52].

## 4. Conclusions

The positive synergetic effect during co-pyrolysis of lignite/HDPE mixture, which resulted in higher yields of liquid and gaseous products, and smaller yield of solid product, than theoretical ones is clearly observed at a temperature ≥450 °C, with the greatest being at 500 °C. This is also documented by Rock-Eval analysis of pyrolytic solid residues, TGA data, and GC–MS analysis of liquid pyrolysates. The carbon isotopic signatures of individual *n*-alkanes and *n*-alkenes, being enriched in ^12^C, in lignite/HDPE liquid co-pyrolysates in relation to both lignite and HDPE liquid pyrolysates unambiguously confirmed the synergetic effect at 450 °C and particularly at 500 °C, which promotes degradation of both HDPE and kerogen, associated with preference cleavage of more labile ^12^C–^12^C bonds.

The results indicate that co-pyrolysis of lignite and HDPE notably increases the content of gaseous (~1.7 times) and liquid products (up to 15 times), and improves the quality of liquid and solid products, in comparison with pyrolysis of lignite alone under the same conditions. The composition of liquid lignite/HDPE co-pyrolysis product at 500 °C, as documented by bulk composition and GC–MS analysis of individual hydrocarbons, is comparable to those of crude oil of terrestrial origin. Elemental analysis and Rock-Eval pyrolysis showed that the co-pyrolysis solid residues contain a higher amount of organic carbon than initial lignite and solid products of lignite pyrolysis, which has a positive influence on the calorific value. Net calorific values of lignite/HDPE solid residues are greater compared with lignite and similar to those of sub-bituminous coal and highly volatile bituminous coal. The gaseous product obtained by lignite/HDPE co-pyrolysis has a higher amount of hydrocarbons than that obtained by pyrolysis of lignite alone. Concerning the estimated lignite/HDPE co-pyrolysis gas composition, based on TGA-FTIR, comprising mainly of lower hydrocarbons, CO, and CO_2_, it may serve for heating.

Data obtained from pyrolysis of HDPE alone indicate that it cannot be concurrent to well-developed catalytic thermal processes for its recycling. However, concerning the huge amount of produced HDPE, at least part of this plastic material can be reused for advanced treatment of lignite, which can replace environmentally non-friendly household combustion, particularly in countries where this low-rank coal represents the main source of energy.

## Figures and Tables

**Table 1:** The yields of pyrolysis products obtained in open system pyrolysis and differences between experimental and theoretical (calculated) yields (%, wt.).

Sample	Liquid Product	Gaseous Product	Solid Residue
Lignite 400	1.37	19.34	79.29
Lignite 450	1.44	23.87	74.69
Lignite 500	1.57	25.02	73.41
Lignite 550	1.26	26.60	72.14
Lignite 600	1.13	29.39	69.48
HDPE 400	12.96	7.41	79.63
HDPE 450	21.32	11.51	67.17
HDPE 500	33.94	15.77	50.29
HDPE 550	33.29	48.84	17.87
HDPE 600	15.49	71.18	13.33
Lignite/HDPE 400	7.57	14.00	78.43
Lignite/HDPE 450	17.83	22.23	59.94
Lignite/HDPE 500	26.34	42.34	31.32
Lignite/HDPE 550	24.58	45.01	30.41
Lignite/HDPE 600	13.08	58.06	28.86
Lignite/HDPE 400 (T)	7.17	13.38	79.45
Lignite/HDPE 450 (T)	11.38	17.69	70.93
Lignite/HDPE 500 (T)	17.75	20.40	61.85
Lignite/HDPE 550 (T)	17.28	37.72	45.01
Lignite/HDPE 600 (T)	8.31	50.29	41.41
	**Difference: Experimental yield—Theoretical yield (%, wt.)**		
**Temperature (^o^C)**	**Liquid product**	**Gaseous product**	**Solid residue**
400	0.40	0.62	−1.02
450	6.45	4.54	−10.99
500	8.59	21.94	−30.53
550	7.31	7.29	−14.60
600	4.77	7.78	−12.55

**Legend:** Lignite 400—pyrolysis of lignite at 400 °C; Lignite 450—pyrolysis of lignite at 450 °C; Lignite 500—pyrolysis of lignite at 500 °C; Lignite 550—pyrolysis of lignite at 550 °C; Lignite 600—pyrolysis of lignite at 600 °C; high-density polyethylene (HDPE) 400—pyrolysis of HDPE at 400 °C; HDPE 450—pyrolysis of HDPE at 450 °C; HDPE 500—pyrolysis of HDPE at 500 °C; HDPE 550—pyrolysis of HDPE at 550 °C; HDPE 600—pyrolysis of HDPE at 600 °C; Lignite/HDPE 400—pyrolysis of lignite/HDPE mixture at 400 °C (experimental yield); Lignite/HDPE 450—pyrolysis of lignite/HDPE mixture at 450 °C (experimental yield); Lignite/HDPE 500—pyrolysis of lignite/HDPE mixture at 500 °C (experimental yield); Lignite/HDPE 550—pyrolysis of lignite/HDPE mixture at 550 °C (experimental yield); Lignite/HDPE 600—pyrolysis of lignite/HDPE mixture at 600 °C (experimental yield); Lignite/HDPE 400 (T)—theoretical (calculated) yield of lignite/HDPE mixture pyrolysis at 400 °C; Lignite/HDPE 450 (T)—theoretical (calculated) yield of lignite/HDPE mixture pyrolysis at 450 °C; Lignite/HDPE 500 (T)—theoretical (calculated) yield of lignite/HDPE mixture pyrolysis at 500 °C; Lignite/HDPE 550 (T)—theoretical (calculated) yield of lignite/HDPE mixture pyrolysis at 550 °C; Lignite/HDPE 600 (T)—theoretical (calculated) yield of lignite/HDPE mixture pyrolysis at 600 °C; theoretical (calculated) yield of lignite/HDPE mixture pyrolysis = (yield of lignite pyrolysis + yield of HDPE pyrolysis)/2.

**Table 2:** Rock-Eval data of initial substrates (lignite, HDPE, and their mixture) and solid residues obtained in open system pyrolysis.

Sample	S1	S2	S3	T_max_	TOC	HI	OI	PI
Lignite	0.80	29.75	14.83	384	29.34	101.40	50.55	0.026
HDPE	3.19	892.37	0.95	471	83.17	1072.95	1.14	0.004
Lignite/HDPE	2.11	473.27	8.17	458	55.48	853.05	14.73	0.004
Lignite 400	0.57	5.53	9.84	449	29.97	18.45	32.83	0.093
Lignite 450	0.49	3.66	6.27	522	32.57	11.24	19.25	0.118
Lignite 500	0.40	1.88	4.09	569	36.58	5.14	11.18	0.175
HDPE 400	2.59	636.68	0.67	454	85.94	740.84	0.78	0.004
HDPE 450	2.13	353.00	0.25	453	86.07	410.13	0.29	0.006
HDPE 500	3.18	177.02	0.11	456	86.16	205.45	0.13	0.018
Lignite/HDPE 400	1.29	315.65	6.48	462	56.77	556.02	11.41	0.004
Lignite/HDPE 450	0.87	119.53	4.98	458	59.88	199.62	8.32	0.007
Lignite/HDPE 500	1.13	35.25	2.87	456	62.08	56.78	4.62	0.031
Lignite/HDPE (T)	2.00	461.06	7.89	427.5	56.26	587.17	25.84	0.015
Lignite/HDPE 400 (T)	1.58	321.11	5.26	451.5	57.96	379.65	16.81	0.049
Lignite/HDPE 450 (T)	1.31	178.33	3.26	487.5	59.32	210.68	9.77	0.062
Lignite/HDPE 500 (T)	1.79	89.44	2.10	512.5	61.37	105.29	5.65	0.097

**Legend:** Lignite—initial lignite; HDPE—initial HDPE; Lignite/HDPE—mixture of initial lignite and initial HDPE (mass ratio, 1:1) (experimental data); Lignite/HDPE (T)—mixture of initial lignite and initial HDPE (mass ratio, 1:1) (theoretical/calculated data); S1—free hydrocarbons (mg HC/g sample); S2—pyrolysate hydrocarbons (mg HC/g sample); HC—hydrocarbons; S3—amount of CO_2_ generated from oxygenated functional groups (mg CO_2_/g sample); T_max_—temperature corresponding to S2 peak maximum(°C); TOC—total organic carbon (%, wt.); HI—hydrogen index = (S2 × 100)/TOC (mg HC/g TOC); OI—oxygen index = (S3 × 100)/TOC (mg CO_2_/g TOC); PI—production index = S1/(S1 + S2). For other abbreviations, see the legend of Table 1.

**Table 3:** Results of elemental analysis of initial substrates (lignite, HDPE, and their mixture) and solid residues obtained in open system pyrolysis (%, wt.).

Sample	Carbon	C_org_	Hydrogen	Nitrogen	Sulphur	Oxygen	Ash
Lignite	31.32	29.77	2.79	0.61	0.80	17.36	47.12
HDPE	84.9	84.90	14.70	/	/	0.40	
Lignite/HDPE	57.03	56.33	8.90	0.36	0.44	10.10	23.17
Lignite 400	31.83	30.33	1.79	0.56	0.77	15.81	49.24
Lignite 450	34.99	33.48	1.52	0.59	0.73	12.72	49.45
Lignite 500	38.56	37.02	1.41	0.53	0.72	9.06	49.72
Lignite 550	40.75	39.23	1.25	0.53	0.74	6.76	49.97
Lignite 600	41.67	40.17	1.09	0.57	0.77	5.72	50.18
HDPE 400	86.22	86.22	13.60	/	/	0.18	/
HDPE 450	86.26	86.26	13.59	/	/	0.15	/
HDPE 500	86.37	86.37	13.55	/	/	0.08	/
HDPE 550	86.55	86.55	13.43	/	/	0.02	/
HDPE 600	86.67	86.67	13.32	/	/	0.01	/
Lignite/HDPE 400	58.01	57.21	6.37	0.33	0.39	9.57	25.33
Lignite/HDPE 450	61.62	60.83	4.31	0.35	0.30	7.75	25.67
Lignite/HDPE 500	66.50	65.69	3.58	0.29	0.28	3.22	26.13
Lignite/HDPE 550	67.48	66.73	3.03	0.31	0.29	2.61	26.28
Lignite/HDPE 600	67.92	67.27	2.78	0.35	0.31	2.08	26.56
Lignite/HDPE (T)	58.11	57.34	8.75	0.31	0.40	8.88	23.56
Lignite/HDPE 400 (T)	59.03	58.28	7.70	0.28	0.39	7.99	24.62
Lignite/HDPE 450 (T)	60.63	59.87	7.56	0.30	0.37	6.44	24.73
Lignite/HDPE 500 (T)	62.47	61.70	7.48	0.27	0.36	4.57	24.86
Lignite/HDPE 550 (T)	63.65	62.89	7.34	0.27	0.37	3.39	24.99
Lignite/HDPE 600 (T)	64.17	63.42	7.21	0.29	0.39	2.86	25.09

**Legend:** C_org_—content of total organic carbon, obtained by elemental analysis; oxygen = 100—(carbon + hydrogen + nitrogen + sulphur + ash); theoretical values are adjusted at two decimal places.

**Table 4:** Calculated net calorific values (MJ/kg) of solid residues of lignite and lignite/HDPE mixtures obtained in open system pyrolysis.

Sample	Q_n1_	Q_n2_	Q_n3_	Q_n4_	Q_n5_	Q_n6_	Q_n7_	Q_n8_	Q_n9_	Q_n10_
Lignite	11.87	11.50	11.88	10.87	11.42	10.29	11.85	12.48	10.95	11.84
Lignite 400	11.38	10.69	11.03	10.16	10.60	9.73	11.25	12.15	10.18	11.24
Lignite 450	12.69	11.91	12.16	11.40	11.81	11.00	12.45	13.04	11.47	12.44
Lignite 500	14.35	13.55	13.70	13.04	13.44	12.66	14.01	14.14	13.20	13.99
Lignite 550	15.29	14.46	14.54	13.95	14.34	13.59	14.88	14.77	14.16	14.87
Lignite 600	15.62	14.74	14.80	14.24	14.62	13.90	15.18	14.99	14.47	15.16
Lignite/HDPE 400	25.93	26.09	26.23	25.00	25.97	23.86	25.42	22.87	25.73	25.41
Lignite/HDPE 450	25.76	25.09	25.17	24.19	24.94	23.32	24.95	23.03	24.75	24.93
Lignite/HDPE 500	27.48	26.56	26.51	25.71	26.39	24.94	26.47	24.25	26.32	26.45
Lignite/HDPE 550	27.45	26.32	26.25	25.52	26.14	24.82	26.37	24.30	26.09	26.35
Lignite/HDPE 600	27.46	26.25	26.17	25.47	26.07	24.81	26.35	24.32	26.03	26.33

**Legend**: Q_n_—net calorific value; Q_n1_ = 38.2 × mass proportion of C + 84.9 × (mass proportion of H − mass proportion of O/8)—0.62 [53]; Q_n2_ = 34.1 × mass proportion of C + 121.4 × mass proportion of H − 15.3 × mass proportion of O + 10.5 × mass proportion of S [53]; Q_n3_ = 33.9 × mass proportion of C + 121.5 × mass proportion of H − 12.7 × mass proportion of O + 9.3 × mass proportion of S [53]; Q_n4_ = 33.4 × mass proportion of C + 117.7 × mass proportion of H − 15.6 × mass proportion of O [54]; Q_n5_ = 33.8 × mass proportion of C + 122.3 × (mass proportion of H − mass proportion of O/8) + 9.4 × mass proportion of S [55]; Q_g_—gross calorific value; Q_g6_ = 33 × mass proportion of C + 120 × mass proportion of H − 16 × mass proportion of O [41]; Q_n7_ = 35.2 × mass proportion of C + 116.2 × mass proportion of H + 6.3 x mass proportion of N + 10.5 x mass proportion of S − 11.1 × mass proportion of O [40,56]; Q_g8_ = 971 + 323.6 × %C + 711 × %H [57]; Q_g9_ = (145.44 × %C + 620.28 × %H + 40.5 × %S − 77.54 x%O)/0.429923 [53,55]; Q_g10_ = (151.2 × %C + 499.77 × %H + 45 ×%S − 47.7 × %O + 27 × %N)/0.429923 [53,58]; Q_n_ = Q_g_ − 21.96 × mass proportion of H [53,59].

**Table 5:** Bulk composition of lignite extractable organic matter (EOM) and the liquid pyrolysis products (%, wt.) obtained in open system pyrolysis.

Sample	Aliphatic Hydrocarbons	Aromatic Hydrocarbons	NSO + Asphaltenes	Total Hydrocarbons
Lignite EOM	7.1	5.2	87.7	12.3
Lignite 400	12.9	11.1	76.0	24.0
Lignite 450	14.8	12.3	72.9	27.1
Lignite 500	16.3	14.6	69.1	30.9
HDPE 400	100	0	0	100
HDPE 450	100	0	0	100
HDPE 500	100	0	0	100
Lignite/HDPE 400	40.2	15.3	44.5	55.5
Lignite/HDPE 450	48.5	18.7	32.8	67.2
Lignite/HDPE 500	52.3	20.4	27.3	72.7
Lignite/HDPE 400 (T)	56.45	5.55	38.00	62.00
Lignite/HDPE 450 (T)	57.40	6.15	36.45	63.55
Lignite/HDPE 500 (T)	58.15	7.30	34.55	65.45

**Legend**: NSO—polar fraction, containing nitrogen, sulphur, and oxygen compounds.

**Table 6:** Contents of individual compounds (%) in the aliphatic fraction of the liquid pyrolysis products.

Compound	Lignite 400	Lignite 450	Lignite 500	HDPE 400	HDPE 450	HDPE 500	Lignite/HDPE 400	Lignite/HDPE 450	Lignite/HDPE 500
C_12_	/	/	/	1.09	1.71	0.24	/	/	/
C_13_	/	/	/	2.02	2.65	1.77	/	/	0.77
C_14_	/	/	/	2.75	3.47	3.64	/	0.41	2.97
C_15_	0.13	/	/	3.19	3.96	4.69	0.57	1.74	5.05
C_16_	1.64	0.06	0.11	3.27	3.98	4.87	1.92	2.72	5.37
Norpristane	1.26	0.12	0.14	/	/	/	/	/	/
C_17_	4.77	1.63	1.53	3.45	3.97	5.06	3.11	3.28	5.38
Pristane	1.26	0.37	0.30	/	/	/	/	/	/
Pristene	4.77	1.58	1.88	/	/	/	/	/	/
C_18_	5.34	5.24	2.25	3.58	4.08	5.21	3.89	3.73	5.13
Phytane	0.70	0.60	0.45	/	/	/	/	/	/
C_19_	6.34	7.31	6.95	3.72	3.97	5.10	4.43	4.02	4.79
C_20_	6.58	8.80	7.64	3.80	3.94	4.85	4.64	4.05	4.56
C_21_	6.53	8.90	8.55	3.77	3.77	4.29	4.42	4.09	4.34
C_22_	7.31	9.29	9.75	4.03	3.78	4.26	4.68	4.19	4.27
C_23_	6.69	8.54	8.67	4.01	3.73	3.94	4.46	4.08	4.49
C_24_	8.38	8.47	9.34	4.30	3.94	4.02	4.59	4.21	4.44
C_25_	7.42	8.09	9.33	4.25	3.82	3.83	4.46	4.45	4.26
C_26_	7.84	7.79	8.12	4.41	3.99	3.83	4.54	4.38	4.13
C_27_	5.56	5.82	6.63	4.25	3.84	3.57	4.36	4.47	4.33
C_28_	4.83	4.39	5.25	4.63	3.86	3.68	4.81	4.76	4.12
C_27_ α	0.36	0.26	0.88	/	/	/	/	/	/
C_27_ β	1.43	1.02	2.22	/	/	/	/	/	/
C_29_	3.61	3.53	2.34	4.18	3.70	3.44	4.59	4.69	3.86
C_29_ αβ	0.90	0.36	0.98	/	/	/	/	/	/
C_30_	1.90	2.05	0.98	4.37	3.95	3.58	4.70	4.60	3.72
C_29_ βα	0.80	0.64	1.41	/	/	/	/	/	/
C_30_ αβ	0.16	0.11	0.58	/	/	/	/	/	/
C_31_	1.69	1.88	1.20	4.17	3.69	3.24	4.42	4.39	3.45
C_29_ ββ	0.28	0.31	0.71	/	/	/	/	/	/
C_30_ βα	0.19	0.19	0.53	/	/	/	/	/	/
C_32_	0.77	0.99	0.64	4.36	3.86	3.42	4.65	4.30	3.28
C_33_	0.55	1.13	0.63	4.05	3.64	3.14	4.22	4.11	2.88
C_34_	/	0.53	/	3.88	3.61	2.95	4.25	3.95	2.59
C_35_	/	/	/	3.40	2.93	2.44	3.65	3.62	2.21
C_36_	/	/	/	2.91	2.63	2.14	3.52	3.37	1.90
C_37_	/	/	/	2.23	2.18	1.87	2.72	2.95	1.54
C_38_	/	/	/	1.53	1.81	1.61	2.67	2.61	1.50
C_39_	/	/	/	1.25	1.40	1.37	1.95	2.28	1.14
C_40_	/	/	/	0.98	1.27	1.15	1.74	2.06	1.19
C_41_	/	/	/	1.00	1.15	0.98	1.22	1.34	0.86
C_42_	/	/	/	0.64	0.99	1.13	0.71	0.86	0.92
C_43_	/	/	/	0.56	0.72	0.68	0.12	0.28	0.57

**Legend**: C_x_ designates *n*-alkane + terminal *n*-alkene + terminal diene; x represents total number of carbon atoms. For other assignments, see the legend of Figure S1.

**Table 8:** Contents of individual compounds (%) in aromatic fraction of the liquid pyrolysis products.

Compound	Lignite 400	Lignite 450	Lignite 500	Lignite/HDPE 400	Lignite/HDPE 450	Lignite/HDPE 500
DBF	3.41	3.06	3.74	0.02	0.10	0.05
ΣTMN	24.22	18.22	14.41	5.00	6.95	2.85
F	3.17	3.86	4.20	0.88	2.13	1.54
ΣMDBF	8.73	9.86	8.91	3.00	4.63	3.45
ΣTeMN	19.39	16.19	14.09	21.16	9.11	9.80
Cadalene	4.11	3.92	2.98	1.99	1.30	1.07
ΣMF	6.83	8.51	8.67	3.15	12.16	10.54
P	3.11	3.90	5.30	4.18	5.78	6.60
A	1.67	2.10	2.28	1.87	2.07	1.84
ΣMP	6.87	7.11	9.29	14.48	16.02	18.31
ΣDMP	11.52	11.36	10.77	19.82	16.83	16.18
Flu	2.34	2.55	2.58	4.90	3.56	4.21
Py	0.75	1.21	1.56	2.19	2.64	3.39
Retene	0.97	1.16	1.16	/	/	/
ΣMPy	2.91	3.83	6.41	9.12	8.42	13.46
B[a]A	/	0.95	1.20	1.94	1.63	2.09
C	/	0.83	1.06	2.16	2.65	2.10
ΣMC	/	1.39	1.38	4.12	4.02	2.53

**Legend:** For compound assignments, see the legend of Figure S5.

**Table 9:** Values of common organic geochemical naphthalene and phenanthrene parameters in liquid products obtained in open system pyrolysis.

Sample	TMNR	TeMNR	MPI 1	MPI 3	PAI 1
Lignite EOM	N.D.	N.D.	N.D.	N.D.	N.D.
Lignite 400	0.09	0.12	0.62	0.79	1.96
Lignite 450	0.17	0.16	0.53	0.89	1.27
Lignite 500	0.21	0.17	0.45	1.01	0.85
HDPE 400	N.D.	N.D.	N.D.	N.D.	N.D.
HDPE 450	N.D.	N.D.	N.D.	N.D.	N.D.
HDPE 500	N.D.	N.D.	N.D.	N.D.	N.D.
Lignite/HDPE 400	0.18	0.29	0.61	0.78	1.96
Lignite/HDPE 450	0.26	0.38	0.64	0.99	1.49
Lignite/HDPE 500	0.38	0.39	0.64	1.16	1.26

**Legend**: N.D.—not determined owing to the absence of naphthalene and phenanthrene derivatives in the lignite EOM (Figure S5a) and HDPE liquid pyrolysates; TMNR = 1,3,7-TMN/(1,3,7-TMN + 1,2,5-TMN) [72]; TeMNR = 2,3,6,7-TeMN/1,2,3,6-TeMN [73]; TMNR and TeMNR ratios are calculated from the typical *m/z* 170 and 184 mass fragmentograms of aromatic fraction (Figure S7); MPI 1 = 1.5 × (2-MP + 3-MP)/(1-MP + 9-MP + P) [74]; MPI 3 = (2-MP + 3-MP)/(1-MP + 9-MP) [75]; PAI 1 = (2-MP + 3-MP + 1-MP + 9-MP)/P [76]; MPI 1, MPI 3, and PAI 1 ratios are calculated from the typical *m/z* 178 and 192 mass fragmentograms of aromatic fraction (Figure S7); TMN—trimethylnaphthalene; TeMN—tetramethylnaphthalene; P—phenanthrene; MP—methylphenanthrene. For other abbreviations, see the legends of Table 1 and Table 2.

## Data Availability

Not applicable.

## Supplementary Materials

The following are available online at https://www.mdpi.com/2073-4360/13/5/759/s1. Figure S1: The total ion chromatograms (TICs) of aliphatic fractions of lignite EOM (a), and liquid products obtained by lignite pyrolysis in the open system at 400 ^o^C (b), 450 ^o^C (c) and 500 ^o^C (d)., Figure S2: TICs of liquid products obtained by HDPE pyrolysis in the open system at 400 ^o^C (a), 450 ^o^C (b) and 500 ^o^C (c), Figure S3: TICs of aliphatic fractions of liquid products obtained by lignite/HDPE co-pyrolysis in the open system at 400 ^o^C (a), 450 ^o^C (b) and 500 ^o^C (c), Figure S4: Mass fragmentograms (*m/z* 71) of aliphatic fractions of liquid products obtained by pyrolysis in the open system at 500 ^o^C; lignite (a), HDPE (b), lignite/HDPE mixture (c), Figure S5: TICs of aromatic fractions of lignite EOM (a), and liquid products obtained by lignite pyrolysis in the open system at 400 ^o^C (b), 450 ^o^C (c) and 500 ^o^C (d), Figure S6: TICs of aromatic fractions of liquid products obtained by lignite/HDPE co-pyrolysis in the open system at 400 ^o^C (a), 450 ^o^C (b) and 500 ^o^C (c), Figure S7: Mass fragmentograms *m/z* (170 + 184 and 178 + 192) of aromatic fractions of liquid products obtained by pyrolysis in the open system at 500 ^o^C; lignite (a, c), lignite/HDPE mixture (b, d), Figure S8: TGA-FTIR thermograms of lignite (a), HDPE (b) and lignite/HDPE mixture (c), representing absorbance with respect to time and wavenumber. A time of 20 minutes corresponds to a temperature of 227 ^o^C, 40 minutes to 427 ^o^C, 60 minutes to 627 ^o^C, 80 minutes to 827 ^o^C. The ramp rate was 10 ^o^C/min.

## References

[1] Strategy Energy Development of the Republic of Serbia until 2015. http://www.smeits.rs/include/data/docs0066.doc.

[2] Đoković N., Mitrović D., Životić D., Bechtel A., Sachsenhofer R.F., Matić V., Glamočanin L., Stojanović K. (2018). Petrographical and organic geochemical study of the lignite from the Smederevsko Pomoravlje field (Kostolac Basin, Serbia). Int. J. Coal Geol..

[3] Mitrović D., Đoković N., Životić D., Bechtel A., Šajnović A., Stojanović K. (2016). Petrographical and organic geochemical study of the Kovin lignite deposit, Serbia. Int. J. Coal Geol..

[4] Jelenković R., Kostić A., Životić D., Ercegovac M. (2008). Mineral resources of Serbia. Geol. Carpath..

[5] Reichl C., Schatz M., Zsak G. (2016). World-Mining-Data. Minerals Production.

[6] Serbia, the Country Of Lignite. https://naukakrozprice.rs/srbija-zemlja-lignita.

[7] (2019). Electric Power Industry of Serbia, The Technical Yearbook. http://www.eps.rs/cir/SiteAssets/Pages/tehnicki-izvestaji/TEH_Godisnjak2019_web_s.pdf.

[8] European Commission (2015). Improvement of Coal Carbonization through the Optimization of Fuel in Coking Coal Blends (RATIO-COAL).

[9] Mitrović D., Đoković N., Životić D., Bechtel A., Cvetković O., Stojanović K. (2017). Characterisation of lignite lithotypes from the “Kovin” deposit (Serbia)–Implications from petrographic, biomarker and isotopic analysis. J. Serb. Chem. Soc..

[10] Li Q., Jiang J., Zhang Q., Zhou W., Cai S., Duan L., Ge S., Hao J. (2016). Influences of coal size, volatile matter content, and ad-ditive on primary particulate matter emissions from household stove combustion. Fuel.

[11] Zhang Y., Schauer J.J., Zhang Y., Zeng L., Wei Y., Liu Y., Shao M. (2008). Characteristics of Particulate Carbon Emissions from Real-World Chinese Coal Combustion. Environ. Sci. Technol..

[12] WHO Guidelines for Indoor Air Quality: Household fuel combustion. https://www.who.int/airpollution/guidelines/household-fuel-combustion/recommendation3/en.

[13] Bond T.C., Covert D.S., Kramlich J.C., Larson T.V., Charlson R.J. (2002). Primary particle emissions from residential coal burning: Optical properties and size distributions. J. Geophys. Res. Space Phys..

[14] Sahu M., Peipert J., Singhal V., Yadama G.N., Biswas P. (2011). Evaluation of Mass and Surface Area Concentration of Particle Emissions and Development of Emissions Indices for Cookstoves in Rural India. Environ. Sci. Technol..

[15] Das D., Dutta S., Bhandarkar U., Sethi V. (2019). Assessment of carbonization of coal as a potential strategy to reduce emissions for domestic applications. Atmos. Pollut. Res..

[16] Xu Y., Zhang Y., Wang Y., Zhang G., Chen L. (2013). Gas evolution characteristics of lignite during low-temperature pyrolysis. J. Anal. Appl. Pyrolysis.

[17] You Q., Wu S.Y., Wu Y.Q., Huang S., Gao J.S., Shang J.X., Min X.J., Zheng H.A. (2017). Product distributions and characteri-zations for integrated mild-liquefaction and carbonization of low rank coals. Fuel Process. Technol..

[18] Makaremi M., Yousefi H., Cavallaro G., Lazzara G., Goh C.B.S., Lee S.M., Solouk A., Pasbakhsh P. (2019). Safely Dissolvable and Healable Active Packaging Films Based on Alginate and Pectin. Polymers.

[19] Parres F., Peydro M.A., Juarez D., Arrieta M.P., Aldas M. (2020). Study of the Properties of a Biodegradable Polymer Filled with Different Wood Flour Particles. Polymers.

[20] Kumar S., Singh R.K. (2011). Recovery of hydrocarbon liquid from waste high density polyethylene by thermal pyrolysis. Braz. J. Chem. Eng..

[21] Park J.W., Kim J.H., Seo G. (2002). The effect of pore shape on the catalytic performance of zeolites in the liquid-phase degradation of HDPE. Polym. Degrad. Stab..

[22] Punkkinen H., Oasmaa A., Luntama L.J., Nieminen M., Ylijoki L.J. (2017). Thermal Conversion of Plastic Containing Waste: A Review. Research report no D4.1–22.

[23] Sharratt P.N., Lin Y.H., Garforth A.A., Dwyer J. (1997). Investigation of the Catalytic Pyrolysis of High-Density Polyethylene over a HZSM-5 Catalyst in a Laboratory Fluidized-Bed Reactor. Ind. Eng. Chem. Res..

[24] Kim S., Lee N., Lee J. (2020). Pyrolysis for Nylon 6 Monomer Recovery from Teabag Waste. Polymers.

[25] Tissot B.P., Welte D.H. (1984). Petroleum Formation and Occurrence.

[26] Peters K.E., Walters C.C., Moldowan J.M. (2005). The Biomarker Guide, Volume 1: Biomarkers and Isotopes in the Environment and Human History.

[27] Behar F., Kressmann S., Rudkiewicz J., Vandenbroucke M. (1992). Experimental simulation in a confined system and kinetic modelling of kerogen and oil cracking. Org. Geochem..

[28] Shao X., Pang X., Li M., Qian M., Hu T., Li Z., Zhang H., Xu Y. (2020). Hydrocarbon retention in lacustrine shales during thermal maturation: Insights from semi-open system pyrolysis. J. Pet. Sci. Eng..

[29] Vuković N., Životić D., Mendonça Filho J.G., Stevović K.T., Vidó H.M., Mendonça J.O., Stojanović K. (2016). The as-sessment of maturation changes of humic coal organic matter–insights from closed-system pyrolysis experiments. Int. J. Coal Geol..

[30] Monthioux M., Landais P., Durand B. (1986). Comparison between extracts from natural and artificial maturation series of Mahakam delta coals. Org. Geochem..

[31] Monthioux M., Landais P., Monin J.C. (1985). Comparison between natural and artificial maturation series of humic coals from the Mahakam delta, Indonesia. Org. Geochem..

[32] Cai J., Wang Y., Zhou L., Huang Q. (2008). Thermogravimetric analysis and kinetics of coal/plastic blends during co-pyrolysis in nitrogen atmosphere. Fuel Process. Technol..

[33] Matali S., Rahman N.A., Idris S.S., Alias A., Mohatar M. (2015). Co-Pyrolysis and Characteristics of Malaysian Sub-Bituminous Coal and Waste Hdpe Blends Via Tga. J. Teknol..

[34] Ishaq M., Ahmad I., Shakirullah M., Khan M.A., Rehman H.U., Bahader A. (2006). Pyrolysis of some whole plastics and plastics–coal mixtures. Energy Convers. Manag..

[35] Sharypov V.I., Beregovtsova N.G., Kuznetsov B.N., Cebolla V.L., Collura S., Finqueneisel G., Zimny T., Weber J.V. (2007). In-fluence of reaction parameters on brown coal–polyolefinic plastic co-pyrolysis behavior. J. Anal. Appl. Pyrolysis.

[36] Vivero L., Barriocanal C., Álvarez R., Díez M.A. (2005). Effects of plastic wastes on coal pyrolysis behavior and the structure of semicokes. J. Anal. Appl. Pyrolysis.

[37] Zhou L., Luo T., Huang Q. (2009). Co-pyrolysis characteristics and kinetics of coal and plastic blends. Energy Convers. Manag..

[38] Chunmei Q., Min Z., Jianghong W., Puhai Y., Xu Y. (2014). Pyrolysis and co-pyrolysis of lignite and plastic. Int. J. Min. Sci.Technol..

[39] Kríz V., Bičáková O. (2011). Hydrogen from the two-stage pyrolysis of bituminous coal/waste plastics mixtures. Int. J. Hydrogen Energy.

[40] Kathiravale S., Yunus M.N.M., Sopian K., Samsuddin A., Rahman R. (2003). Modeling the heating value of municipal solid waste. Fuel.

[41] Han J., Yao X., Zhan Y., Oh S.Y., Kim L.H., Kim H.J. (2017). A method for estimating higher heating value of biomass-plastic fuel. J. Energy Inst..

[42] Kojic I., Bechtel A., Kittinger F., Stevanovic N., Obradovic M., Stojanovic K. (2018). Study of pyrolysis of high density polyethylene in the open system and estimation of its capability for co-pyrolysis with lignite. J. Serb. Chem. Soc..

[43] Stojanović K., Životić D., Šajnović A., Cvetković O., Nytoft H.P., Scheeder G. (2012). The upper Miocene Drmno lignite field (Kostolac Basin, Serbia): Origin and palaeoenvironmental implications from petrological and organic geochemical studies. J. Serb. Chem. Soc..

[44] Ercegovac M., Kostic A., Karg H., Welte D.H., Littke R. (2003). Temperature and Burial History Modelling of the Drmno and Markovac Depressions, Se Pannonian Basin, Serbia. J. Pet. Geol..

[45] Đoković N., Mitrović D., Životić D., Španić D., Čorbić T.T., Cvetković O., Stojanović K. (2015). Preliminary organic ge-ochemical study of lignite from the Smederevsko Pomoravlje field (Kostolac Basin, Serbia)–Reconstruction of geological evolution and potential for rational utilization. J. Serb. Chem. Soc..

[46] Khedri S., Elyasi S. (2016). Kinetic analysis for thermal cracking of HDPE: A new isoconversional approach. Polym. Degrad. Stab..

[47] Coplen T.B. (2011). Guidelines and recommended terms for expression of stable-isotope-ratio and gas-ratio measurement results. Rapid Commun. Mass Spectrom..

[48] Espitalie J., De Roo G., Marquis F. (1985). La pyrolyse Rock-Eval et ses applications. Deuxième partie. Rev. Inst. Français Pétrole.

[49] ISO 1171 (1997). Methods for Analysis and Testing of Coal and Coke. Determination of Ash Content.

[50] Sun Y.H., Bai F.T., Lü X.S., Li Q., Liu Y.M., Guo M.Y., Guo W., Liu B.C. (2015). A Novel Energy-Efficient Pyrolysis Process: Self-pyrolysis of Oil Shale Triggered by Topochemical Heat in a Horizontal Fixed Bed. Sci. Rep..

[51] Yaari A.M., Dubdub I. (2020). Application of Artificial Neural Networks to Predict the Catalytic Pyrolysis of HDPE Using Non-Isothermal TGA Data. Polymers.

[52] Ye C.P., Yang Z.J., Li W.Y., Rong H.L., Feng J. (2017). Effect of adjusting coal properties on HulunBuir lignite pyrolysis. Fuel Process. Technol..

[53] Hosokai S., Matsuoka K., Kuramoto K., Suzuki Y. (2016). Modification of Dulong’s formula to estimate heating value of gas, liquid and solid fuels. Fuel Process. Technol..

[54] Demirbaş A. (2001). Relationships between lignin contents and heating values of biomass. Energy Convers. Manag..

[55] Lowry H.H. (1947). Chemistry of Coal Utilization.

[56] Annamalai K., Sweeten J.M., Ramalingam S.C. (1987). Technical Notes: Estimation of Gross Heating Values of Biomass Fuels. Trans. ASAE.

[57] Toscano G., Pedretti E.F. (2009). Calorific Value Determination of Solid Biomass Fuel by Simplified Method. J. Agric. Eng..

[58] Boie W. (1953). Fuel technology calculations. Energietechnik.

[59] Tsiamis D.A., Castaldi M.J. (2016). Determining Accurate Heating Values of Non-Recycled Plastics (NRP).

[60] Jamradloedluk J., Lertsatitthanakorn C. (2014). Characterization and Utilization of Char Derived from Fast Pyrolysis of Plastic Wastes. Procedia Eng..

[61] Taylor G.H., Teichmüller M., Davis A., Diessel C.F.K., Littke R., Robert P. (1998). Organic Petrology.

[62] Životić D. (2018). Geology of Coal.

[63] Wong S., Ngadi N., Abdullah T., Inuwa I. (2015). Current state and future prospects of plastic waste as source of fuel: A review. Renew. Sustain. Energy Rev..

[64] Miskolczi N., Bartha L., Deák G., Jóver B., Kalló D. (2004). Thermal and thermo-catalytic degradation of high-density polyethylene waste. J. Anal. Appl. Pyrolysis.

[65] Abadi A.M.S., Haghighi M.N., Yeganeh H., McDonald A.G. (2014). Evaluation of pyrolysis process parameters on poly-propylene degradation products. J. Anal. Appl. Pyrol..

[66] Bray E.E., Evans E.D. (1961). Distribution of n-paraffins as a clue to recognition of source beds. Geochim. Cosmochim. Acta.

[67] Kumar S., Panda A.K., Singh R. (2011). A review on tertiary recycling of high-density polyethylene to fuel. Resour. Conserv. Recycl..

[68] Leary O.M.H. (1981). Carbon isotope fractionation in plants. Phytochemistry.

[69] Collister J.W., Rieley G., Stern B., Eglinton G., Fry B. (1994). Compound-specific δ13C analysis of leaf lipids from plants with differing carbon dioxide metabolism. Org. Geochem..

[70] Huang Y., Lockheart M.J., Collister J.W., Eglinton G. (1995). Molecular and isotopic biogeochemistry of the Miocene Clarkia For-mation: Hydrocarbons and alcohols. Org. Geochem..

[71] Rieley G., Collister J.W., Stern B., Eglinton G. (1993). Gas chromatography-isotope ratio mass spectrometry dioxide metabolisms. Rapid Commun. Mass Spectrom..

[72] Van Aarssen B.G., Bastow T.P., Alexander R., I Kagi R. (1999). Distributions of methylated naphthalenes in crude oils: Indicators of maturity, biodegradation and mixing. Org. Geochem..

[73] George S.C., Lisk M., Summons R.E., Quezada A.R., Horsfield B., Radke M., Schaefer R.G., Wilkes H. (1998). Constraining the oil charge history of South Pepper oilfield from the analysis of bearing fluid inclusions. Advances in Organic Geochemistry1997, Part 1.

[74] Radke M., Welte D.H., Willsch H. (1982). Geochemical study on a well in the Western Canada Basin: Relation of the aromatic dis-tribution pattern to maturity of organic matter. Geochim. Cosmochim. Acta.

[75] Radke M., Radke M. (1987). Organic geochemistry of aromatic hydrocarbons. Advances in Petroleum Geochemistry.

[76] Ishiwatari R., Fukushima K. (1979). Generation of unsaturated and aromatic hydrocarbons by thermal alteration of young kerogen. Geochim. Cosmochim. Acta.

[77] Onwudili J.A., Insura N., Williams P.T. (2009). Composition of products from the pyrolysis of polyethylene and polystyrene in a closed batch reactor: Effects of temperature and residence time. J. Anal. Appl. Pyrolysis.

[78] Singh S., Wu C., Williams P.T. (2012). Pyrolysis of waste materials using TGA-MS and TGA-FTIR as complementary characterisation techniques. J. Anal. Appl. Pyrolysis.

[79] Wilkie C.A. (1999). TGA/FTIR: An extremely useful technique for studying polymer degradation. Polym. Degrad. Stab..

[80] Arias M., Penichet I., Ysambertt F., Bauzab R., Zougagh M., Ríos Á. (2009). Fast supercritical fluid extraction of low- and high-density polyethylene additives: Comparison with conventional reflux and automatic Soxhlet extraction. J. Supercrit. Fluids.

