# Supplementary material for: Study of the Synergetic Effect of Co-Pyrolysis of Lignite and High-Density Polyethylene Aiming to Improve Utilization of Low-Rank Coal
Source: Polymers (Basel). 2021 Feb 28;13(5):759. doi: 10.3390/polym13050759 (PMC7957703; doi:10.3390/polym13050759)
Supplement: Supplementary file 1 [file polymers-13-00759-s001.pdf]

polymers

MDPI

Supplement of

Study of the Synergetic Effect of Co-Pyrolysis of Lignite and High-Density Polyethylene Aiming to Improve Utilization of Low-Rank Coal

Ivan Kojić [1], Achim Bechtel [2], Nikoleta Aleksić [3], Dragana Životić [3], Snežana Trifunović [4], Gordana Gajica [5] and Ksenija Stojanović [4,*]

[1] University of Belgrade, Innovation Center of the Faculty of Chemistry, Studentski trg 12-16, 11000 Belgrade, Serbia; ivankojic@chem.bg.ac.rs
[2] Montanuniversität Leoben, Department of Applied Geosciences and Geophysics, Peter-Tunner-Str. 5, A-8700, Leoben, Austria; achim.bechtel@outlook.de
[3] University of Belgrade, Faculty of Mining and Geology, Đušina 7, 11000 Belgrade, Serbia; nikoleta.aleksic@rgf.bg.ac.rs, dragana.zivotic@rgf.bg.ac.rs
[4] University of Belgrade, Faculty of Chemistry, Studentski trg 12-16, 11000 Belgrade, Serbia; snezanat@chem.bg.ac.rs, ksenija@chem.bg.ac.rs
[5] University of Belgrade, Institute of Chemistry, Technology and Metallurgy, Center of Chemistry, Studentski trg 12-16, 11000 Belgrade, Serbia; ggajica@chem.bg.ac.rs
* Correspondence: ksenija@chem.bg.ac.rs and xenasyu@yahoo.com; Tel.: +381 11 3336 776 (K.S.)

2.1. Samples (Short outline of geological settings of the Kostolac Basin)

The Kostolac Basin is located at the southeastern margin of the Pannonian Basin System (about 90 km east of Belgrade) and covers an area of 145 km^2. The Kostolac Basin is divided into three fields, Drmno, Ćirikovac and Smederevsko Pomoravlje. The Drmno field is currently in exploitation, the Ćirikovac open pit is exhausted, while the Smederevsko Pomoravlje field is still under exploration. Lignite resources and reserves of the Kostolac Basin are estimated at 2 billion tonnes, while reserves in the Smederevsko Pomoravlje field are estimated at 355 Mt. Annually, the Kostolac Basin produces 9-12 Mt of lignite [7].

The basement of the Kostolac Basin consists of Devonian crystalline rocks overlain by Neogene sediments [43]. Neogene sediments consist of Lower Miocene clastic syn-rift sediments, up to 2725 m thick that are overlain by Middle Miocene succession (sandstones, sandy claystones, marlstones and clayey limestones, deposited in marine and brackish environments, which thickness exceeds 1200 m [44]. The Upper Miocene succession is divided into Pannonian and Pontian deposits. Pannonian sediments are more than 350 m thick and consist of fresh water sandstone, clay, siltstone and marlstone with two coal seams (up to 6 m and 1 m thick, respectively). Pontian sediments consist of sand, silt, clays and lignite seams deposited in shallow lacustrine, delta plain and fluvial environments. The Pontian coal-bearing series includes five lignite seams (from base to top: seams III, II-a, II, I-a, and I), but only seams III, II and I are considered economic. The average thickness of seam III is 19.4 m, while it is 1.4 m for IIa, 4.1 m for II, 1.5 for Ia and 13.9 m for I coal seam.

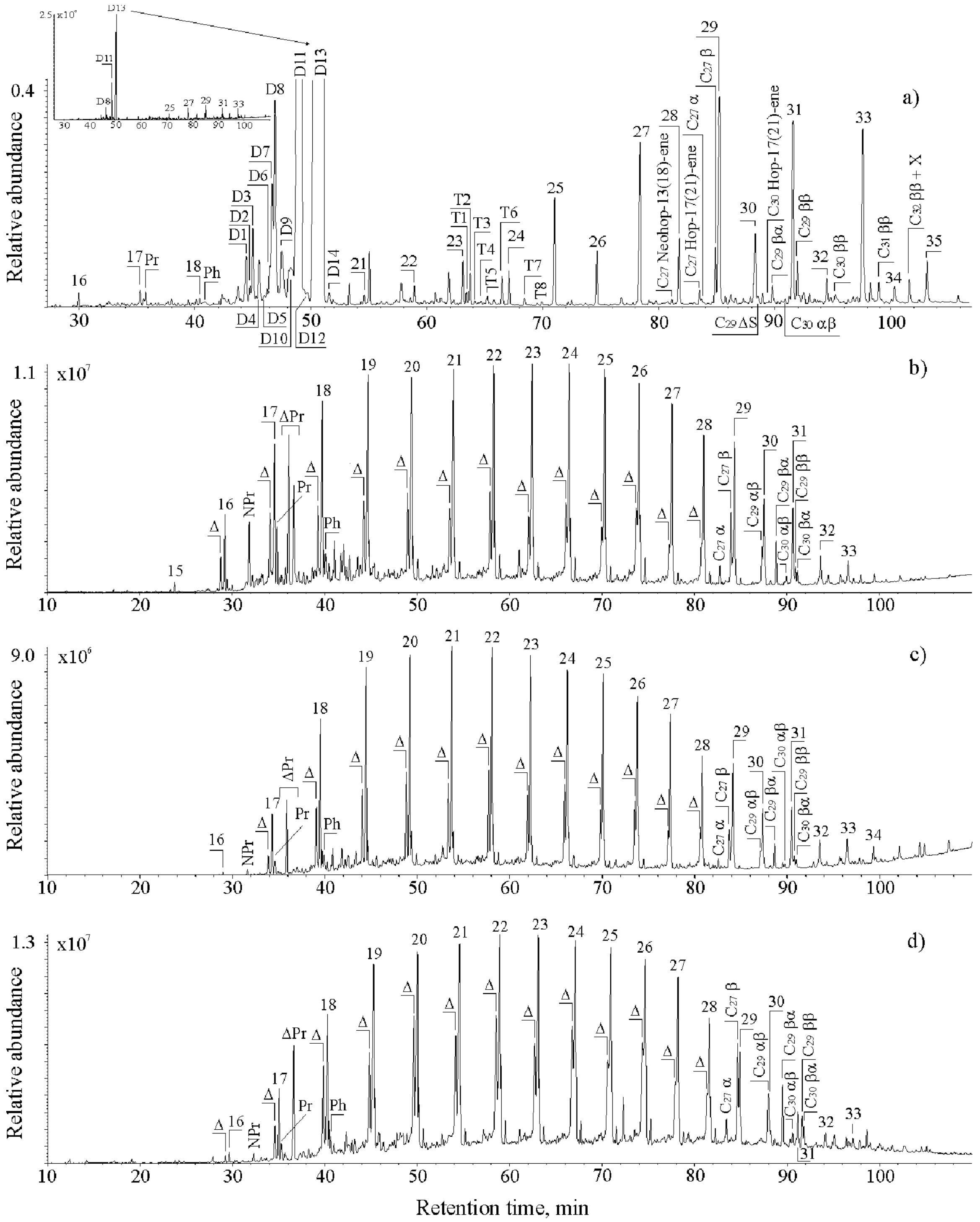

Figure S1. The total ion chromatograms (TICs) of aliphatic fractions of lignite EOM (a), and liquid products obtained by lignite pyrolysis in the open system at 400 °C (b), 450 °C (c) and 500 °C (d). **Legend**: *n*-Alkanes are labelled according to their carbon number; Pr – Pristane; Ph – Phytane; D1 – Isopimaradiene; D2 – 8β(H)-Labdane; D3 – Isopimaradiene; D4 – Norisopimarane; D5 – 8α(H)-Labdane; D6 – Atisene; D7 – Norpimarane; D8 – Beyerane; D9 – Isophyllocladene; D10 – Fichtelite; D11 – Pimarane; D12 – 16β(H)-Phyllocladane; D13 – 16α(H)-Phyllocladane; D14 – 16α(H)-Kaurane; T1 – Des-A-olean-13(18)-ene; T2 – Des-A-olean-12-ene; T3 – Des-A-olean-18-ene + Des-A-urs-13(18)-ene; T4 – Des-A-oleanadiene; T5 – Des-A-urs-12-ene; T6 – Des-A-lupane; T7 – Des-A-triterpene; T8 – Des-A-oleanane; α and β designate configuration at C-17 in C_{27} hopanes; ββ, βα and αβ designate configurations at C-17 and C-21 in C_{29}-C_{32} hopanes; C_{27} neohop-13(18)-ene – C_{27} 22,29,30-trisnorneohop-13(18)-ene; C_{27} hop-17(21)-ene – C_{27} 22,29,30-trisnorhop-17(21)-ene; ΔS – Sterene; X – Unknown compound co-eluting with C_{32} ββ

hopane; Δ –*n*-Alk-1-enes with same number of carbon atoms as *n*-alkanes; NPr – Norpristane; ΔPr – Pristene.

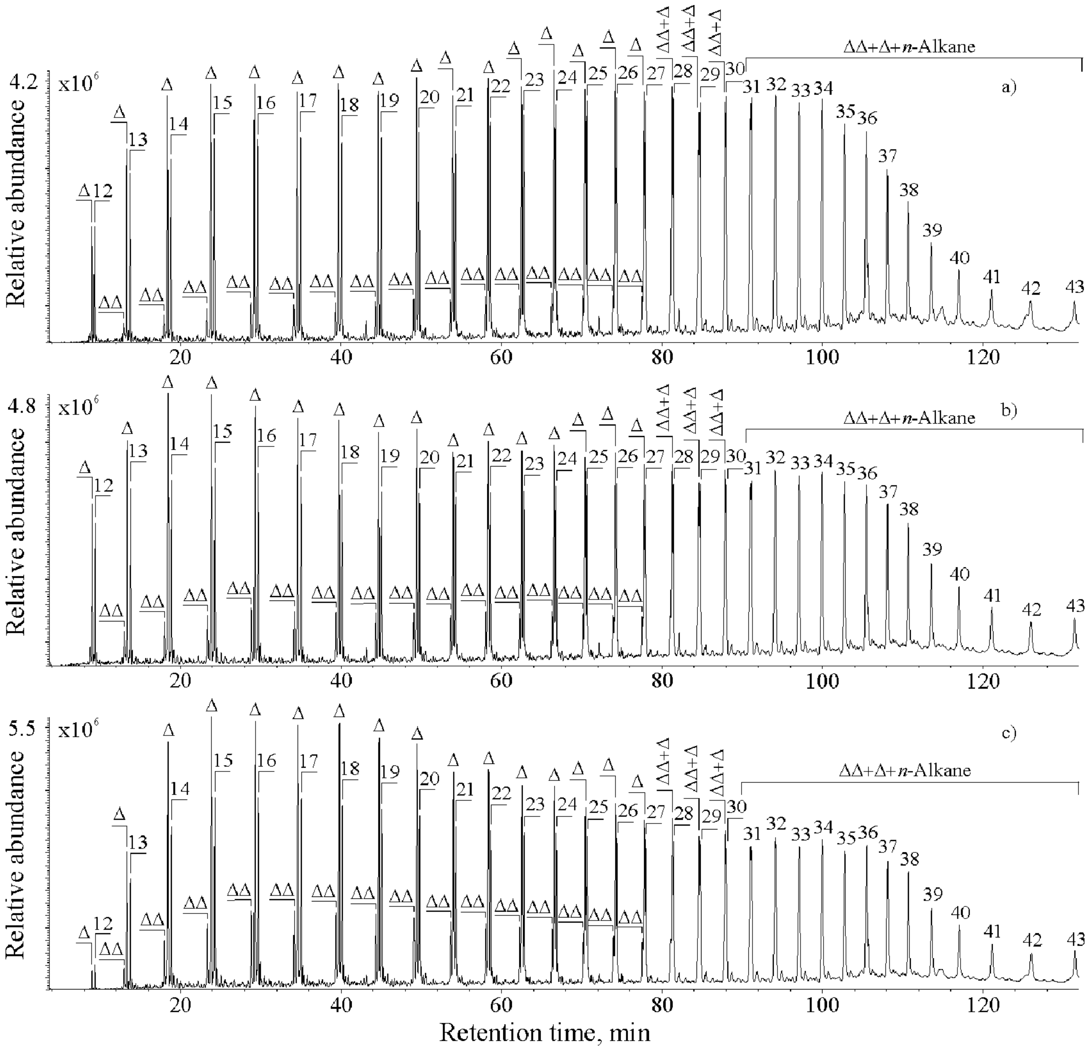

Figure S2. TICs of liquid products obtained by HDPE pyrolysis in the open system at 400 °C (a), 450 °C (b) and 500 °C (c).

<u>**Legend**</u>: *n*-Alkanes are labelled according to their carbon number; Δ – *n*-Alk-1-enes with same number of carbon atoms as *n*-alkanes; ΔΔ – Terminal dienes with same number of carbon atoms as *n*-alkanes.

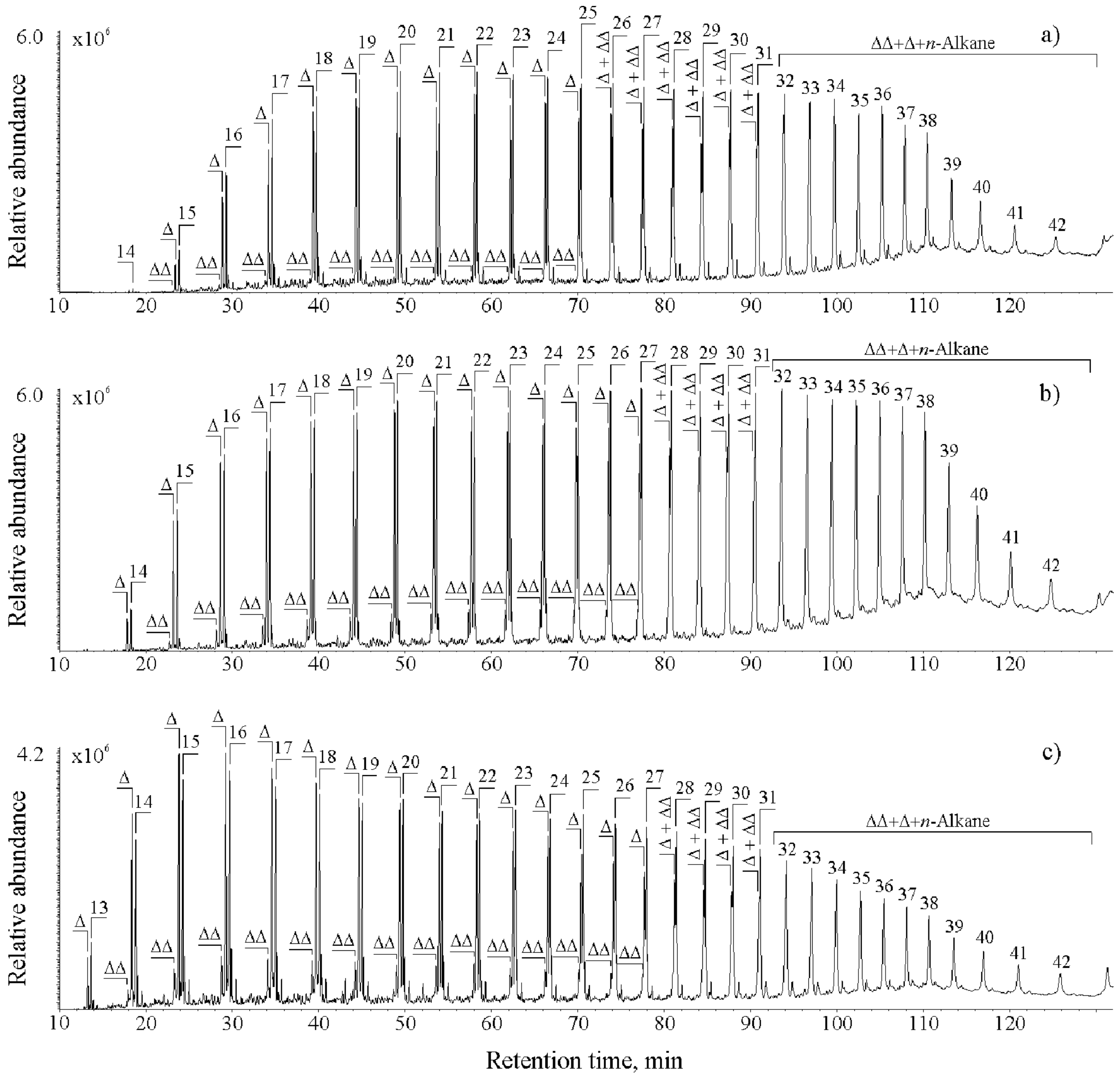

Figure S3. TICs of aliphatic fractions of liquid products obtained by lignite/HDPE co-pyrolysis in the open system at 400 °C (a), 450 °C (b) and 500 °C (c).

<u>Legend:</u> For peak assignments, see the legend of Figure S2.

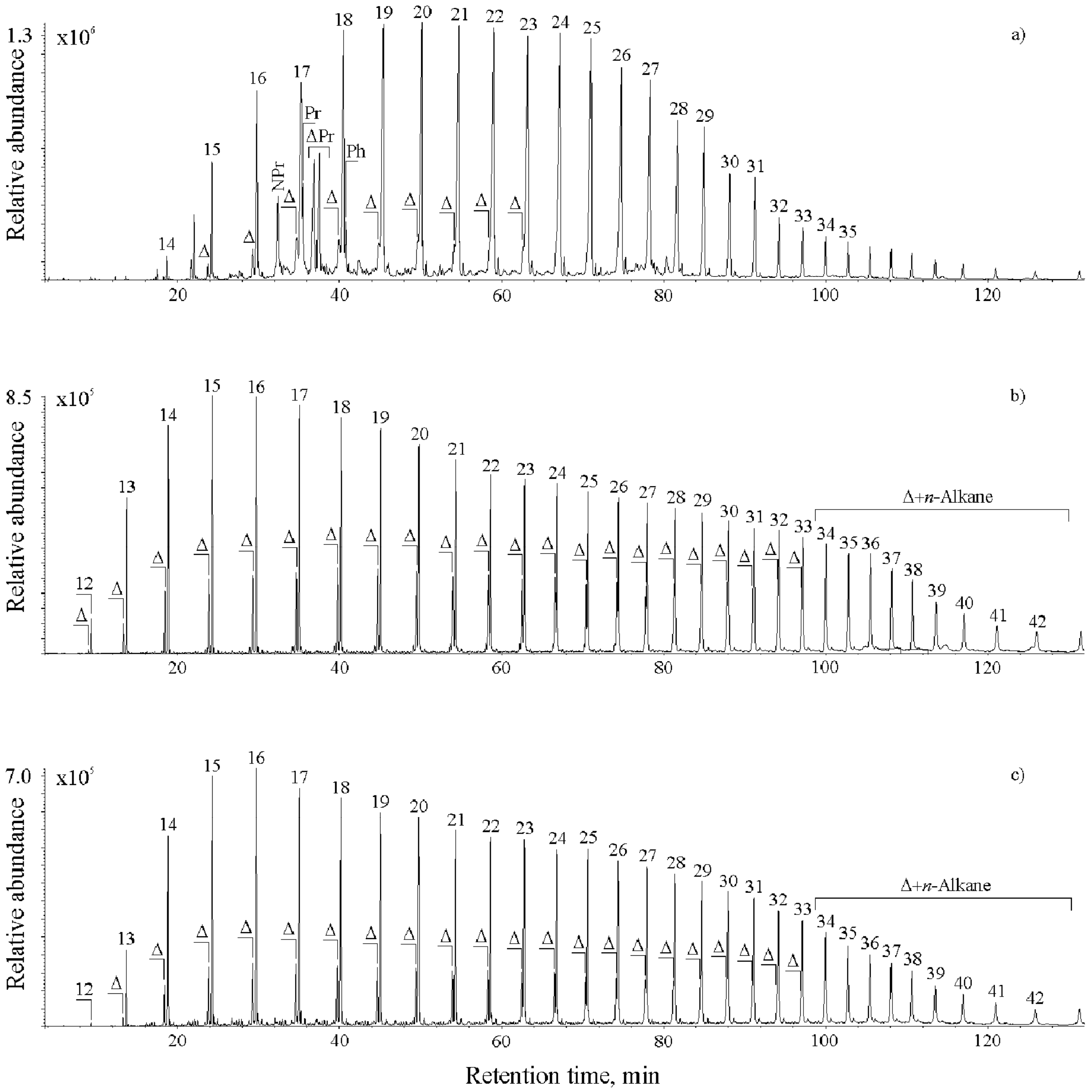

Figure S4. Mass fragmentograms (*m*/*z* 71) of aliphatic fractions of liquid products obtained by pyrolysis in the open system at 500 °C; lignite (a), HDPE (b), lignite/HDPE mixture (c).

<u>**Legend:**</u> For peak assignments, see the legends of Figures S1 and S2.

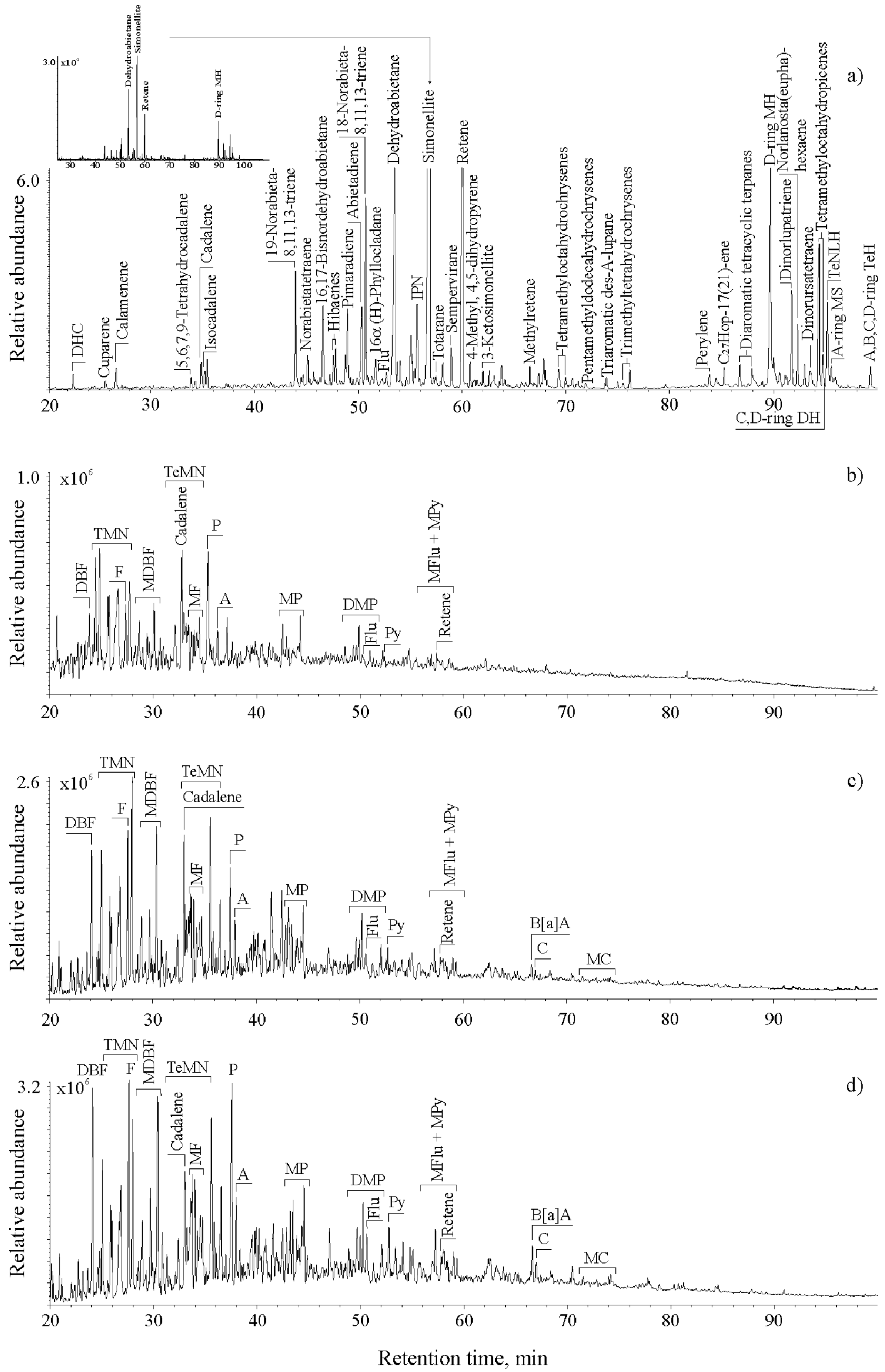

Figure S5. TICs of aromatic fractions of lignite EOM (a), and liquid products obtained by lignite pyrolysis in the open system at 400 °C (b), 450 °C (c) and 500 °C (d).

Legend: DHC – Dihydro-ar-curcumene; Flu – Fluoranthene; IPN – 2-Methyl-1-(4′-methylpentyl), 6-*iso*propylnaphthalene; MH – Monoaromatic hopane; DH – Diaromatic hopane; TeNLH – Tetranor-lupa-1,3,5(10),6,8,11,13-heptaene TeH – Tetraaromatic hopane; DBF – Dibenzofuran; TMN – Trimethylnaphthalene; TeMN – Tetramethylnaphthalene; F – Fluorene; MDBF – Methyldibenzofuran; MF – Methylfluorene, P – Phenanthrene; A – Anthracene; MP – Methylphenanthrene; DMP – Dimethylphenanthrene; Py – Pyrene; MFlu – Methylfluoranthene; MPy – Methylpyrene; C – Chrysene; MC – Methylchrysene; B[a]A – Benz[a]anthracene.

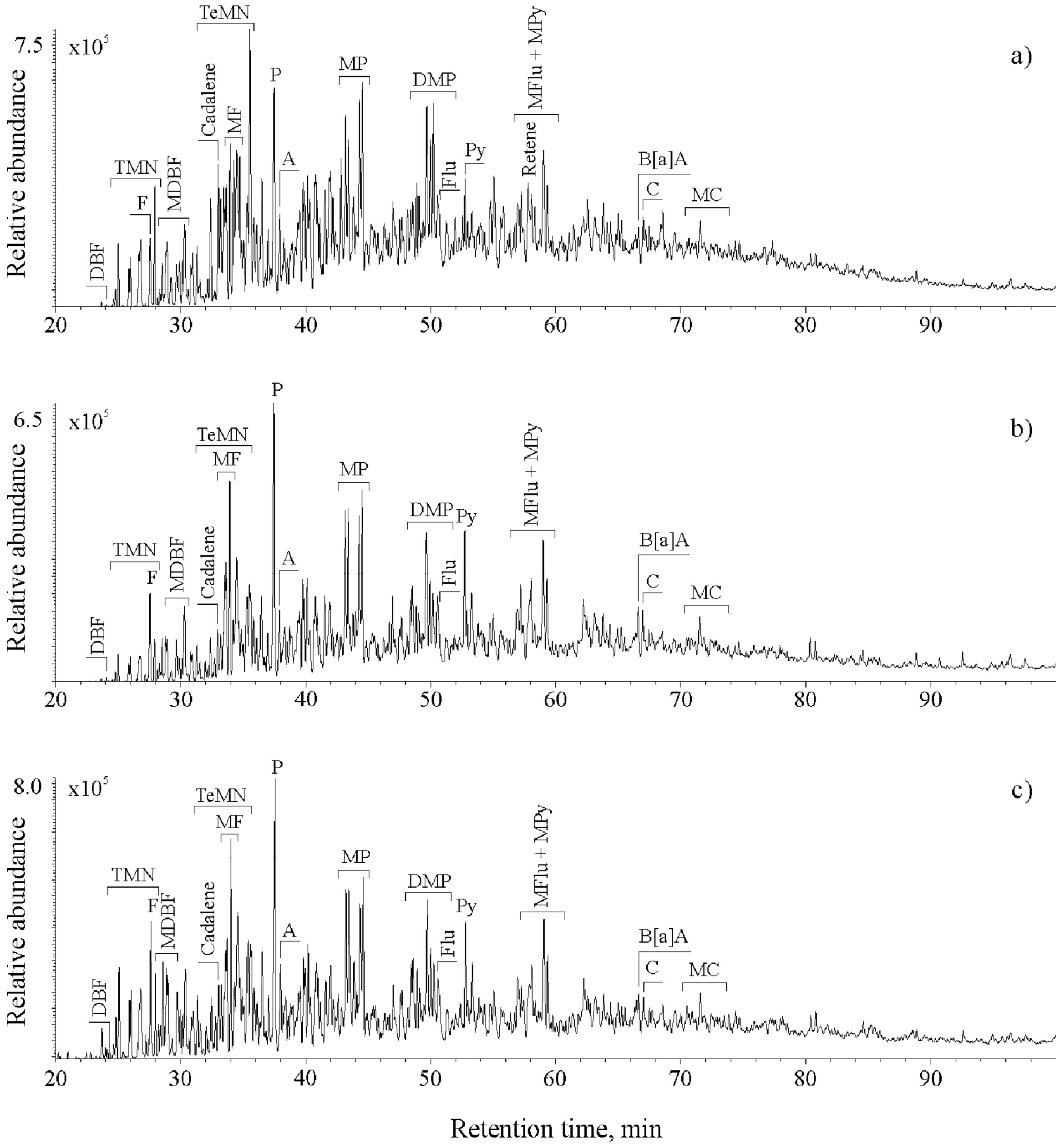

Figure S6. TICs of aromatic fractions of liquid products obtained by lignite/HDPE co-pyrolysis in the open system at 400 °C (a), 450 °C (b) and 500 °C (c).

Legend: For peak assignments, see the legend of Figure S5.

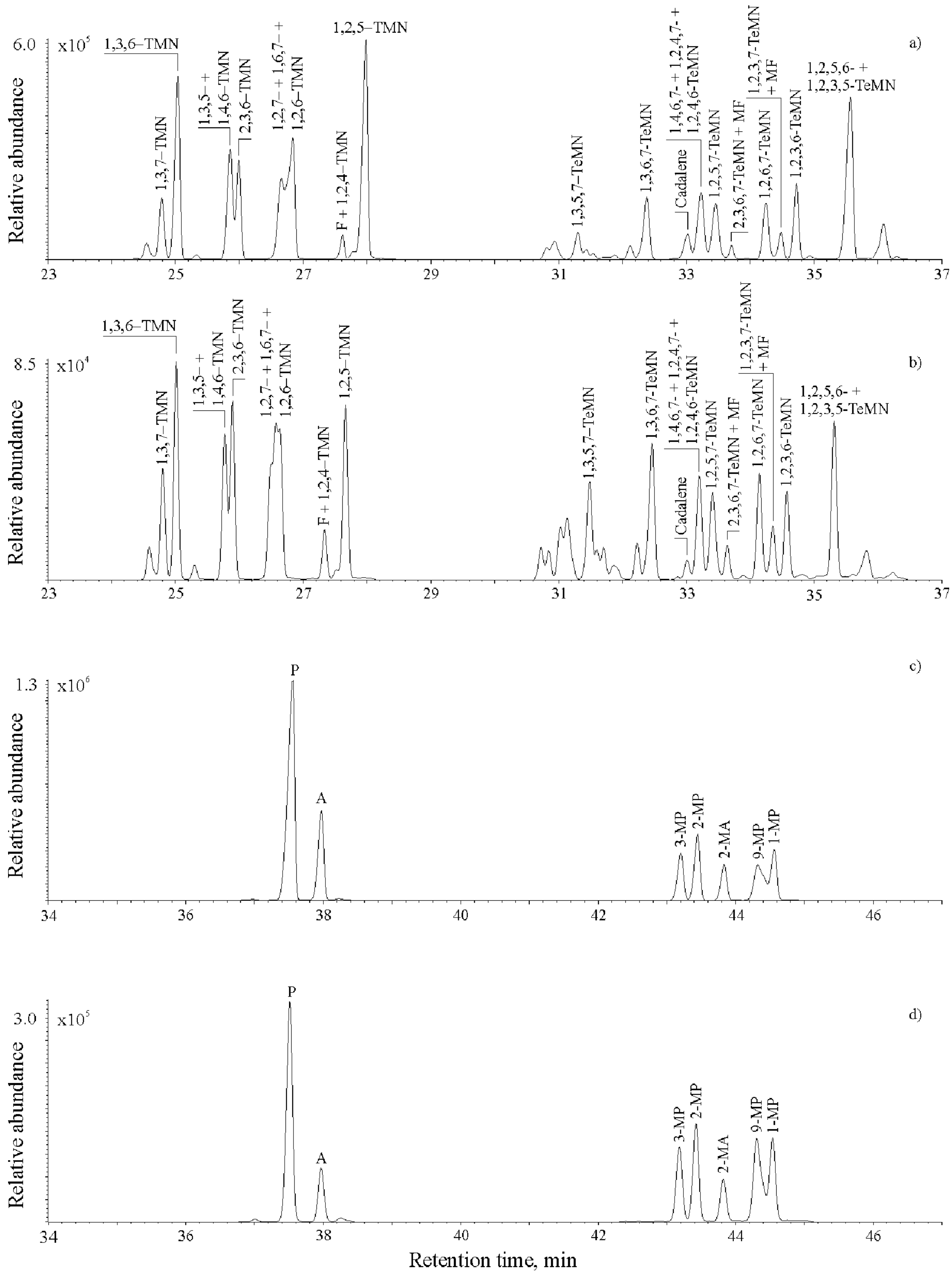

Figure S7. Mass fragmentograms *m*/*z* (170+184 and 178+192) of aromatic fractions of liquid products obtained by pyrolysis in the open system at 500 °C; lignite (a, c), lignite/HDPE mixture (b, d). <u>**Legend**</u>: 2-MA – Methylanthracene; For other peak assignments, see the legend of Figure S5.

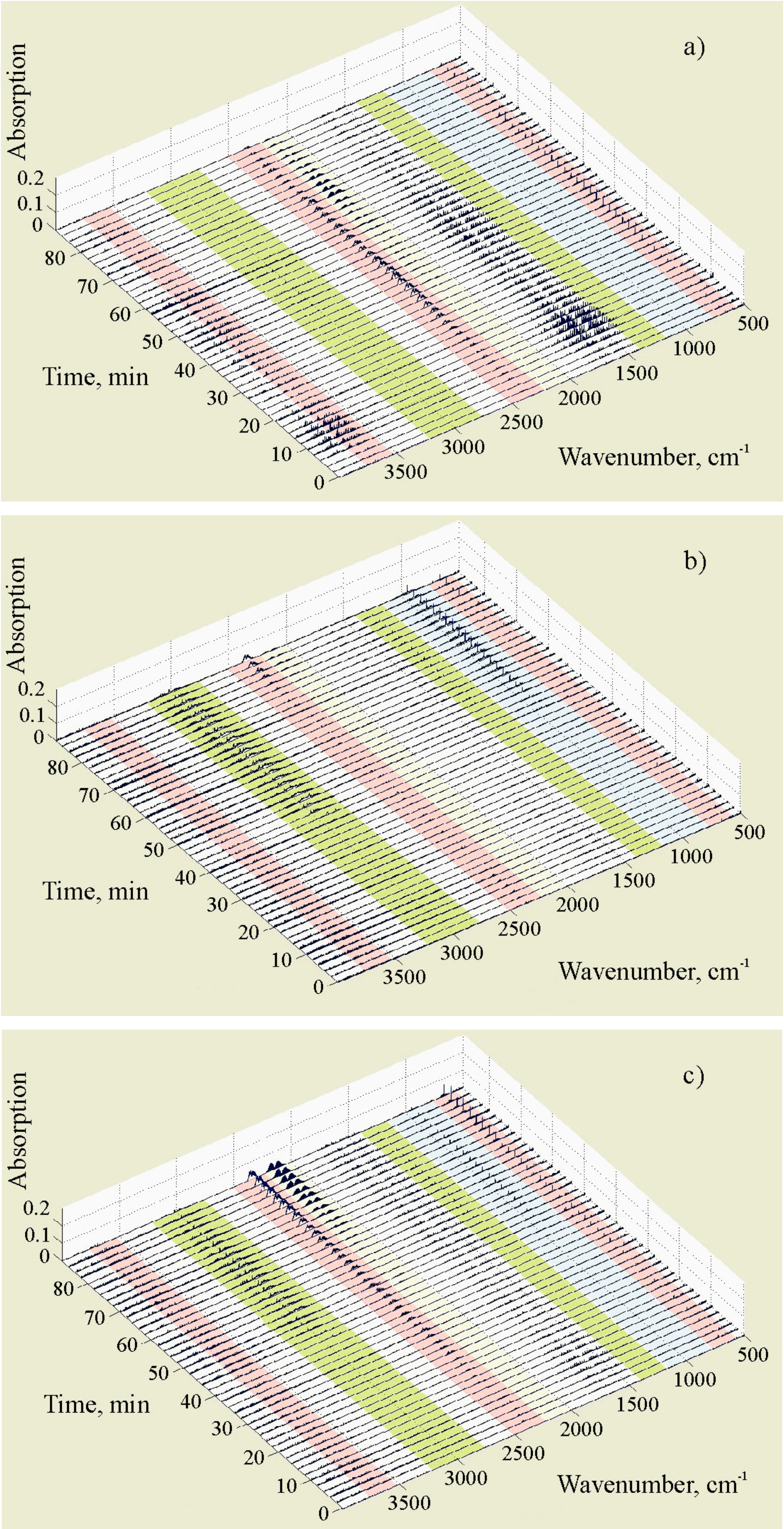

Figure S8. TGA-FTIR thermograms of lignite (a), HDPE (b) and lignite/HDPE mixture (c), representing absorbance with respect to time and wavenumber. A time of 20 minutes corresponds to a temperature of 227 °C, 40 minutes to 427 °C, 60 minutes to 627 °C, 80 minutes to 827 °C. The ramp rate was 10 °C/min.
